# Global estimation of dengue disability weights based on clinical manifestations data

**DOI:** 10.1186/s40249-025-01317-5

**Published:** 2025-06-09

**Authors:** Han-Qi Ouyang, Zi-Yu Zhao, Okugbe Ebiotubo Ohore, Wei-Hao Li, Li Hong, Li-Ying Wang, Hong-Rui Zhang, Fu-Chun Zhang, Raman Velayudhan, Daniel Argaw Dagne, Ibrahima Socé Fall, Guo-Jing Yang

**Affiliations:** 1 School of Public Health, Hainan Medical University (Hainan Academy of Medical Sciences), Haikou, Hainan China; 2 NHC Key Laboratory of Tropical Disease Control, School of Tropical Medicine, Hainan Medical University (Hainan Academy of Medical Sciences), Haikou, Hainan China; 3 Guangzhou Medical Research Institute of Infectious Diseases, Infectious Disease Center, Guangzhou Eighth People’s Hospital, Guangzhou Medical University, Guangzhou, 510440 China; 4 Department of Control of Neglected Tropical Diseases, World Health Organization, Geneva, Switzerland

**Keywords:** Dengue, Clinical manifestation, Disability weight, Meta-analysis, Region-specific

## Abstract

**Background:**

Dengue is a major global health threat with varied clinical manifestations across age groups, countries, and regions. This study aims to estimate global dengue disability weights (DWs) based on clinical manifestations data and examine variations across different demographics and geographical areas. These findings will inform public health strategies and interventions to reduce the global burden of dengue.

**Methods:**

We conducted a systematic search across six databases (Scopus, Web of Science, PubMed, China National Knowledge Infrastructure, Wanfang Data, and Database of Chinese sci-tech periodicals) for studies on human dengue clinical manifestations or infection from the establishment of each database through December 31, 2023. DWs were estimated by combining clinical manifestations frequencies with corresponding DW values derived from the Global Burden of Disease (GBD) study, using Monte Carlo simulations to generate uncertainty intervals. Odds ratios (*OR*s) with 95% confidence intervals (*CI*) and Chi-square tests were performed to compare clinical manifestations between adults and children.

**Results:**

A total of 35 adult studies (7109 cases) and 17 pediatric studies (2996 cases) were analysed. Adults had higher rates of muscle pain (*OR* = 9.18; 95% *CI:* 8.17–10.33) and weak (*OR* = 4.95; 95% *CI* 4.12–5.98). Children showed higher frequencies of decreased appetite (*OR* = 0.12; 95% *CI:* 0.11–0.14) and lymphadenectasis (*OR* = 0.04; 95% *CI:* 0.03–0.06). Severe dengue was more prevalent in children (8.2%) than adults (4.6%). The global DW for universal dengue was 0.3258 in adults and 0.4022 in children, with Indian children showing the highest DW for severe dengue (0.6991) and Chinese adult showing the highest DW for severe dengue (0.7214). Regionally, most studies were from South and Southeast Asia, with India contributing the largest number of publications (80 articles). Additionally, India had the highest dengue disease burden in 2021 (352,468.54 person-years).

**Conclusions:**

These findings reveal important age and regional differences in dengue disease burden. There is a relative lack of research on dengue clinical manifestations in several high-burden countries in the Americas, and these gaps may affect the comprehensiveness and accuracy of global dengue disability weight estimates. These highlight the urgent need for targeted interventions and optimized resource allocation to mitigate its global impact.

**Graphical Abstract:**

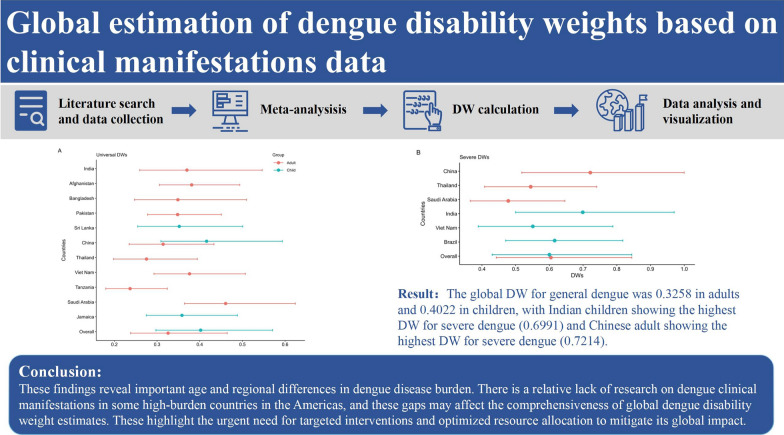

**Supplementary Information:**

The online version contains supplementary material available at 10.1186/s40249-025-01317-5.

## Background

Dengue, a viral infection transmitted through the bites of *Aedes* mosquitoes, is a significant global health threat, particularly in tropical and subtropical regions [1]. As of April 30, 2024, the World Health Organization (WHO) reported over 7.6 million dengue cases worldwide, including over 16,000 severe cases and more than 3000 deaths. While a substantial increase in dengue cases has been reported globally in the last five years, this increase has been particularly pronounced in the Region of the Americas, with over 5 million cases during the same period, of which Brazil accounts for more than 80% of the regional total [2]. This alarming trend underscores the importance of implementing effective and sustainable control measures to mitigate the disease's growing impact.

Clinically, dengue typically presents with high fever, rash, muscle pain, and joint pain. Severe cases may progress to life-threatening outcomes such as dengue hemorrhagic fever (DHF) and dengue shock syndrome (DSS), which demand immediate medical intervention [3]. Recognizing the urgency of severe dengue, WHO set a target in 2020 to eliminate dengue-related fatalities globally by 2030, emphasizing the importance of improved prevention, treatment, awareness, surveillance, and healthcare quality [4]. To guide public health interventions, it is critical to quantify disease severity using standardized tools. Disability weights (DWs), a key component of disability-adjusted life year (DALY) calculations, measure disease severity on a scale from 0 (no health loss) to 1 (equivalent to death) [5]. However, the current Global Burden of Disease (GBD) estimates for dengue provide only universal DW values for moderate, severe, and post-dengue chronic fatigue syndrome, failing to account for age- and region-specific variations in disease burden. Additionally, dengue burden assessments in countries such as Thailand, Indonesia, and China have referred to higher DW values from the literature, reaching up to 0.81 [6, 7]. However, recent studies have shown a significant decline in the proportion of severe dengue cases in China, with the severe rate in adults decreasing from 6.6% to 0.9%, and in children from 15.8% to 0.5%. This decline suggests that these previously used DW values may now overestimate the disease burden in certain regions. This discrepancy underscores the need for systematic evaluations of age- and region-specific DWs to better reflect the real-world burden of dengue.

This study aims to address this gap by systematically analyzing dengue clinical manifestations, focusing on age-specific manifestation profiles and regional DWs. Additionally, temporal changes in the incidence of severe dengue are examined to assess progress in disease management over time. These findings aim to guide resource allocation and support the development of targeted interventions for both adult and pediatric populations.

## Methods

### Search strategy and selection criteria

The study follows the Meta-analysis of Observational Studies in Epidemiology (MOOSE) guidelines for reporting meta-analyses of observational studies in epidemiology (Supplementary file 1) [8]. We systematically searched six databases: Scopus, Web of Science, PubMed, China National Knowledge Infrastructure, Wanfang Data, and Database of Chinese sci-tech periodicals. The search was performed from the inception of each database through December 31, 2023. For English databases, the search string was: (dengue OR dengue fever OR dengue hemorrhagic fever OR breakbone fever OR bouquet fever OR chapenonada OR dengue shock syndrome) AND (clinical OR infection OR manifestation). Equivalent terms were used for Chinese databases. The detailed search strategy for each database is provided in Supplementary file 2. COOC 13.9 software (China) [9] was used for deduplication to retain only unique records.

### Eligibility criteria

We included studies that reported clinical manifestations solely attributable to dengue in human subjects, with confirmed cases based on the WHO Guidelines for the Diagnosis, Treatment, Prevention, and Control of Dengue [10]. Confirmed cases were defined as those diagnosed by laboratory methods, including a positive result from PCR, NS1 antigen, or IgM antibody tests. We excluded studies involving comorbidities (e.g., underlying conditions or pregnancy) that could affect clinical presentation, animal or laboratory studies, articles with unavailable full text, and studies with incomplete or erroneous data. Articles published in languages other than English or Chinese were also excluded.

### Study selection and data extraction

All identified records were managed using EndNote X9 (Clarivate, Philadelphia, USA) and imported into Microsoft Excel 2021 (Microsoft, Redmond, USA) for screening. Three authors independently screened titles and abstracts, followed by full-text reviews to assess eligibility. Disagreements were resolved through discussion until consensus was reached. 

According to the 1997 WHO guidelines, dengue was classified into dengue fever, DHF, and DSS [11]. In the revised 2009 guidelines, the classification was simplified into two categories: dengue (with/without warning signs) and severe dengue [10]. While the 2009 update streamlined the classifications to two categories, the previous definitions of DHF and DSS from 1997 were included within the severe dengue category for consistency in our analysis.

Following both guideline versions, three authors independently extracted data from the included studies using a predefined format to ensure consistency. Extracted data included study characteristics (e.g., country, study period, case counts) and clinical manifestations such as fever, headache, myalgia, arthralgia, vomiting, diarrhea, rash, hepatomegaly, splenomegaly, cardiac impairment, pulmonary outcomes, liver impairment, and shock. The studies included in our analysis were descriptive studies, with case information primarily derived from outpatient or inpatient hospital systems. Any discrepancies were resolved through discussion among the authors. Detailed information about the included literature can be found in Supplementary file 3.

The DWs for the clinical manifestations were primarily obtained from the GBD datasets, including GBD 2021 [12] and GBD 2017 [13]. If data were unavailable from these sources, additional literature was consulted.

### Quality assessment

For assessing the methodological quality of cross-sectional studies, we used the evaluation criteria recommended by the Agency for Healthcare Research and Quality (AHRQ) [14]. Two independent authors conducted this assessment, with any discrepancies resolved through consensus and involvement of a third author. The assessment includes 11 items, with "Yes" responses scored as 1 point. Studies scoring 8 points or higher were considered high quality. To ensure the reliability of the findings, only studies scoring 8 points or higher were included in our analysis. Complete quality assessment results are available in Supplementary file 4.

### Statistical analysis

Heterogeneity was assessed using the Q test and *I*^*2*^ statistic. If *P* < 0.05 in the Q-test or *I*^*2*^ > 50%, heterogeneity was considered present, and a random-effects model was applied; otherwise, a fixed-effects model was used [15]. For proportions equal to 0 or 1, the Freeman-Tukey double arcsine transformation was applied to calculate effect sizes. All meta-analysis results include pooled rates and their 95% confidence intervals (*CI*).

To compare the differences in clinical manifestations between adults and children, we calculated odds ratios (*OR*s) and their 95% *CI* based on the raw data extracted from included studies. Chi-square tests were performed in cases where *OR* values could not be calculated (such as when a cell value was zero). Statistical significance was defined as *P* < 0.05 for all analyses. The results of these comparisons are presented in Fig. 2 and Supplementary file 5.

Subgroup meta-analyses were conducted to examine changes in severe dengue incidence rates before and after 2009 for adults and children, with combined rates calculated using random-effects models. We defined participants under the age of 15 years as children and the remainder as adults. To incorporate as much useful information as possible, we established this age grouping for the current study. Temporal changes and subgroup differences are illustrated in Fig. 3.

DWs for universal and severe dengue were calculated by combining manifestations frequencies with corresponding DW values for different manifestations [16]. The total DW was derived using the formula:

$$DW={\sum }_{A=1}^{n}{P}_{outcome A}*{DW}_{outcome A}$$

where P represents the probability or frequency of each clinical manifestation A, DW represents the disability weight for that manifestation, and n denotes the total number of clinical manifestations considered. Monte Carlo simulations (5000 iterations) were conducted to calculate the uncertainty intervals (UI) for the DW estimate, with manifestation frequencies modeled using a beta distribution and DWs using a log-normal distribution. The results are summarized in Table 1 and Supplementary file 5. Years Lived with Disability (YLD) for 2021 were estimated using GBD data, calculated as YLD = number of cases × DW × duration, with duration set to the standard two weeks. Statistical analyses, including meta-analyses and Monte Carlo simulations, were conducted in R 4.2.3 (Lucent Technologies, Jasmine Mountain, USA).

**Table 1:** Overall dengue disability weighs (Dws) by region

Regions	No. of articles	No. of cases	DWs
Adult universal DWs			
South Asia			
Afghanistan	1	15	0.3811 (0.3056–0.4940)
Bangladesh	1	319	0.3488 (0.2471–0.5097)
India	4	1784	0.3700 (0.2594–0.5463)
Pakistan	1	254	0.3482 (0.2777–0.4506)
East Asia			
China	15	2747	0.3142 (0.2347–0.4333)
Southeast Asia			
Thailand	3	833	0.2753 (0.1980–0.3945)
Vietnam	1	143	0.3761 (0.2927–0.5069)
East Africa			
Tanzania	1	428	0.2368 (0.1797–0.3240)
Middle East			
Saudi Arabia	1	91	0.4606 (0.3643–0.6241)
Overall	28	6614	0.3258 (0.2380–0.4644)
Children universal DWs			
South Asia			
Sri Lanka	1	305	0.3520 (0.2545–0.5008)
East Asia			
China	12	2184	0.4160 (0.3090–0.5935)
North America			
Jamaica	1	339	0.3583 (0.2748–0.4881)
Overall	14	2828	0.4022 (0.2972–0.5707)
Adult severe dengue DWs			
East Asia			
China	4	238	0.7214 (0.5181–1.000)
Southeast Asia			
Thailand	2	70	0.5446 (0.4078–0.7404)
Middle East			
Saudi Arabia	1	187	0.4780 (0.3654–0.6460)
Overall	7	495	0.6044 (0.4426–0.8446)
Children severe dengue DWs			
South Asia			
India	1	22	0.6991 (0.5000–0.9702)
Southeast Asia			
Vietnam	1	69	0.5508 (0.3889–0.7884)
South America			
Brazil	1	77	0.6157 (0.4704–0.8179)
Overall	3	168	0.6000 (0.4303–0.8446)

This table presents DW estimates with 95% confidence intervals for universal dengue (11 countries) and severe dengue (6 countries), stratified by age group and region, revealing notable variations. Since the DW values range between 0 and 1, in our Monte Carlo simulation with 5000 iterations, the upper limits for severe dengue DWs in China exceeded 1. To maintain the reasonableness of the results, we have set the upper limit to 1 for this case in our reported values

## Results

### Overview of included studies

This study systematically searched three English databases and three Chinese databases, with a total of 45,628 articles. After screening titles and abstracts, 1552 articles were selected for full-text review. Based on further screening according to case severity types, age range covered in the studies, and quality assessment scores, we included 28 studies [17–44] on adult dengue (6614 cases) and 14 studies [45–58] on pediatric dengue (2828 cases). These studies were used to compare the clinical manifestations of dengue between adults and children globally, observe changes in severe dengue incidence before and after 2009 in both age groups, and estimate the universal DW values for both groups.

**Fig. 1:**
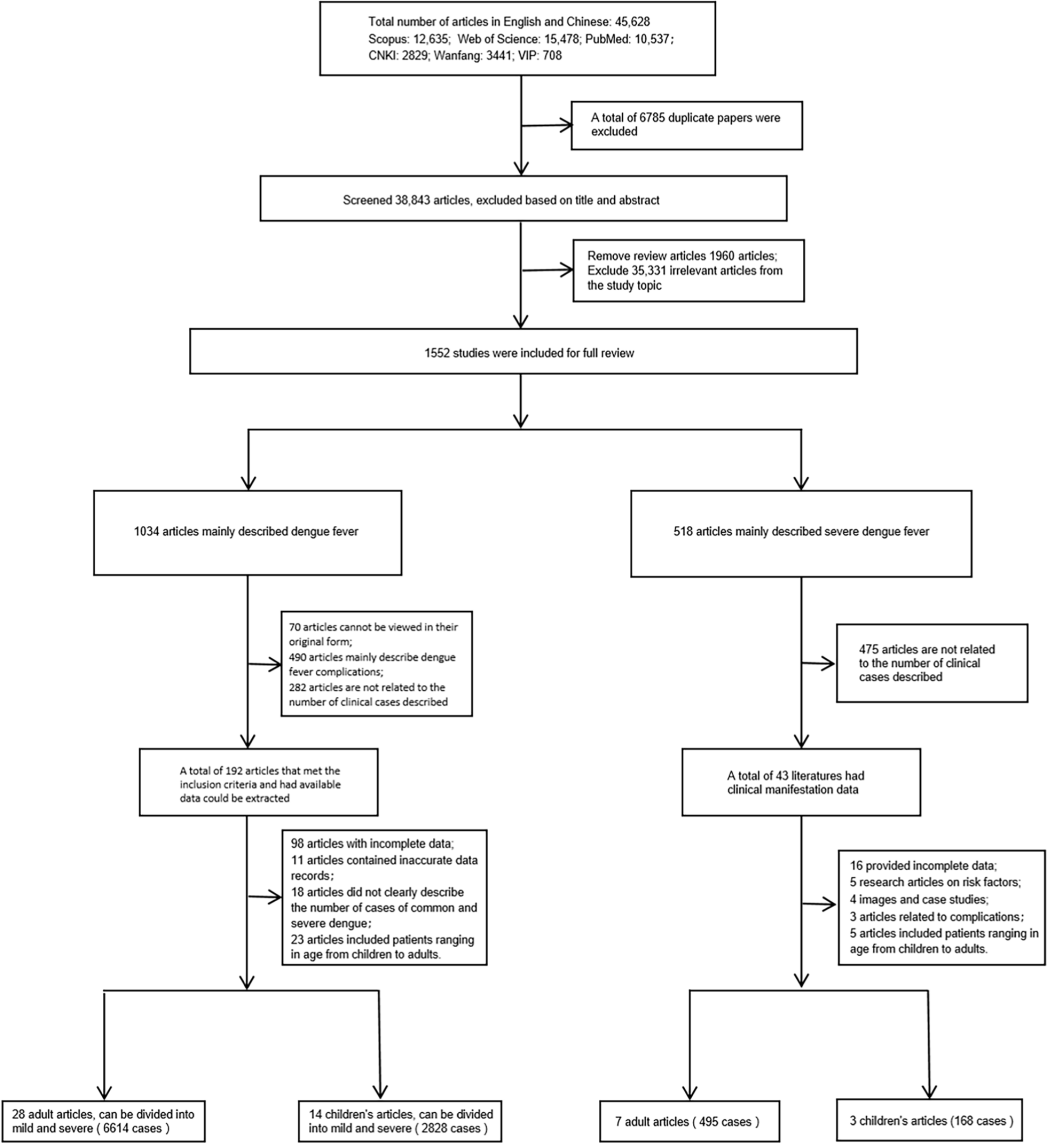
Study selection process flowchart. The flowchart shows the systematic selection and screening of research articles, divided into two paths: articles focusing on dengue (both mild and severe) on the left, and articles focusing only on severe dengue on the right

Additionally, we included studies specifically describing severe dengue, comprising 7 studies [24, 59–64] focused on adults (495 cases) and 3 studies [65–67] focused on children (168 cases), to estimate the DW values for severe dengue in the two age groups. Ultimately, this study utilized data from 52 articles including 24 English-language articles and 28 Chinese-language articles. Figure 1 illustrates the entire literature screening and inclusion process.

### Clinical manifestations of dengue in adults and children worldwide

**Fig. 2:**
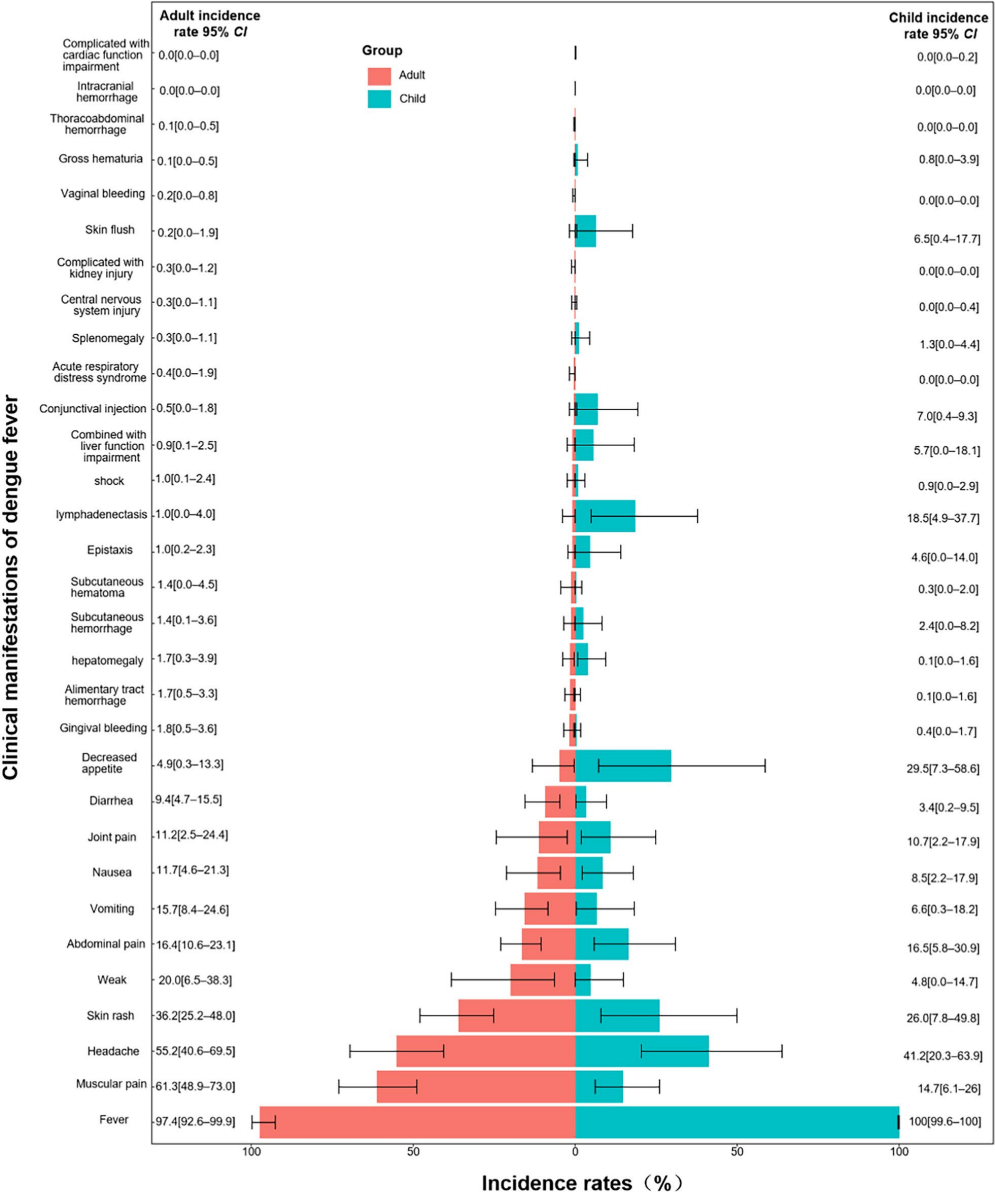
Comparison of clinical manifestations and incidence rates of dengue in adults and children. The bar chart compares the incidence of various clinical manifestations of dengue fever in adults and children, ranked from highest to lowest in the adult group. The Y-axis shows the different clinical manifestations, and the X-axis shows the incidence (%) in both adults and children

Figure 2 displays the incidence rates of various clinical manifestations in dengue patients and provides a comparative analysis between adults (red) and children (blue). *OR* with 95% *CI* and Chi-square tests were used to assess statistical differences between the two groups (details in Supplement file 5). Fever and headache were both highly prevalent in adults and children. Fever was observed in 97.4% of adults and 100.0% of children. Headache occurred in 55.2% of adults and 41.2% of children, with a significant difference (*OR* = 1.76; 95% *CI:* 1.61–1.93). Manifestations more common in adults included muscular pain (*OR* = 9.18; 95% *CI:* 8.17–10.33), skin rash (*OR* = 1.62; 95% *CI:* 1.46–1.78), weak (*OR* = 4.95; 95% *CI:* 4.12–5.98), and diarrhea (*OR* = 2.95; 95% *CI:* 2.37–3.72). Children had higher rates of decreased appetite (*OR* = 0.12; 95% *CI:* 0.11–0.14), skin flush (*OR* = 0.03; 95% *CI:* 0.01–0.05), and lymphadenopathy (*OR* = 0.04; 95% *CI:* 0.03–0.06).

Severe manifestations were more frequent in children, including conjunctival injection (*OR* = 0.07; 95% *CI:* 0.04–0.10), combined with liver function impairment (*OR* = 0.15; 95% *CI:* 0.11–0.21), and epistaxis (*OR* = 0.21; 95% *CI:* 0.15–0.28).

### Temporal changes in global severe dengue comorbidity rates among adults and children

Through subgroup analysis in the meta-analysis, heterogeneity tests were conducted on the comorbidity rates of severe dengue in adults and children over different periods. The results showed significant heterogeneity; therefore, a random-effects model was used to pool the effect sizes. The analysis revealed that the overall severe dengue comorbidity rate in children was 8.20%, higher than the 4.60% observed in adults.

**Fig. 3:**
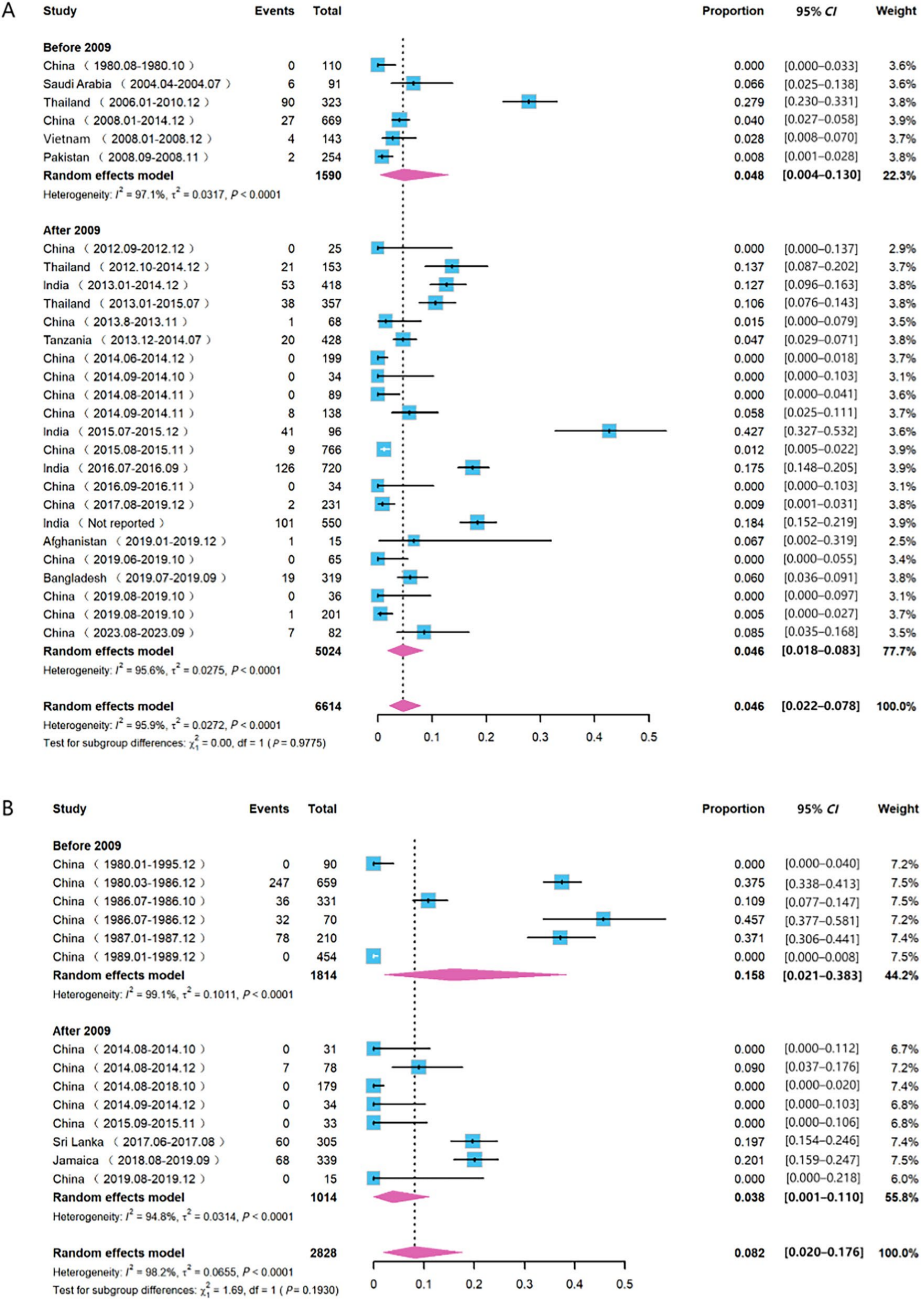
Subgroup analysis of severe dengue combined incidence rate in adults and children. The studies are categorized into adult (**A**) and pediatric (**B**) groups, further divided into before 2009 and after 2009 periods, with a combined incidence rate calculated for each to observe changes between the two time periods

As shown in Fig. 3A, the severe dengue comorbidity rate in adults was 4.80% before 2009, and 4.60% after 2009. The subgroup heterogeneity test indicated no statistically significant difference between the two periods (*P* = 0.98). As shown in Fig. 3B, the severe dengue comorbidity rate in children was 15.80% before 2009 and decreased to 3.80% after 2009. The subgroup heterogeneity test showed no significant difference in the comorbidity rate of severe dengue between the two periods (*P* = 0.19). Although the severe dengue rate in children was not high during the early period, there was a 75.9% reduction in the later period compared to the early period, indicating a significant clinical decline in the severe dengue rate among children.

### Disability weight estimation and uncertainty analysis for dengue cases

The estimation of DW for universal dengue was based on the clinical manifestation data from 6614 adult cases and 2828 pediatric cases. For severe dengue, the DW calculation utilized the clinical data from 495 adult cases and 168 pediatric cases. Supplementary file 5 presents the DWs associated with different manifestations or signs of dengue. These DW values highlight the varying impact of each manifestation or sign on overall health, ranging from mild manifestations such as minor gingival bleeding to severe organ damage. Collecting this information is crucial for accurately estimating the overall disease burden caused by dengue.

Following 5000 Monte Carlo simulations, the range and stability of the DW estimates were validated. In the uncertainty analysis, the universal DW estimate for adult dengue cases was 0.3258, with a 95% UI of 0.2380–0.4644. For pediatric dengue cases, the universal DW estimate was 0.4022, with a 95% UI of 0.2972–0.5707. These results indicate that the universal DW value for children is higher than that for adults. For severe dengue, the DW estimate for adults was 0.6044, with a 95% UI of 0.4426–0.8446, while for children, the DW was 0.6000, with a 95% UI of 0.4303–0.8446.

The universal DW values for adults show Saudi Arabia with the highest value (0.4606), followed by Afghanistan (0.3811), Vietnam (0.3761), and India (0.3700). For children, the highest universal DW values were observed in China (0.4160), followed by Jamaica (0.3583) and Sri Lanka (0.3520). Regarding the DW for severe dengue in adults, China (0.7214) exceeded Thailand (0.5446) and Saudi Arabia (0.4780). For severe dengue in children, India (0.6991) ranked higher than Brazil (0.6157) and Vietnam (0.5508). The uncertainty estimates for universal and severe dengue DWs across different regions are detailed in Table 1.

### Geographical distribution of dengue burden in 2021 and related clinical literature

Supplementary file 6 presents the distribution of dengue-related YLDs across countries in 2021. India had the highest dengue burden, with 352,468.54 person-years, followed by Brazil at 160,741.80 person-years and Pakistan at 32,856.81 person-years. The distribution of dengue-related clinical English-language literature is shown in Supplementary file 7, covering publications from 66 countries. These studies are primarily concentrated in South and Southeast Asia, with India contributing the largest number of publications, totaling 80. Additionally, Thailand contributed 43 studies, and Pakistan contributed 39. In contrast, the number of relevant publications from the Americas, Africa, and Europe was comparatively lower.

## Discussion

### Age-related differences in clinical manifestations and disability weights

Our study reveals notable differences in the clinical presentation of dengue in adult and pediatric patients, which has important implications for disease management and resource allocation. Although fever is common in both adult and pediatric patients, other manifestations present differently. Due to an overreaction of the immune system and tissue damage, adults are more likely to experience manifestations such as muscle pain, headaches, and bleeding-related manifestations. Meanwhile, children are more likely to experience abdominal pain, liver damage, and serious complications such as shock [68]. These differences may be related to the physiological characteristics of different age groups: children's immune systems are not fully developed, and manifestations may be detected late due to limited expression capacity, which increases the risk of developing severe illness. These findings underscore the urgency of implementing specific pediatric dengue management guidelines and strengthening early warning systems.

The meta-analysis revealed a higher overall incidence of severe dengue in pediatric patients (8.2%) compared to adults (4.6%). Since 2009, the incidence of severe dengue has shown different trends across age groups, with adult rates remaining relatively stable (from 4.8% to 4.6%), while pediatric rates have shown a notable decline (from 15.8% to 3.8%). This trend is further exemplified in a meta-analysis of clinical manifestations of dengue in China, where pediatric severe dengue rates dropped from 15.8% in the 1980s to just 0.5% in 2019 [69]. These findings highlight the potential effectiveness of targeted interventions, such as improved access to healthcare services and enhanced surveillance systems, which could be replicated in other dengue-endemic regions [70, 71].

Additionally, the study found age-related differences in DWs, with children exhibiting a higher universal DW (0.4022) than adults (0.3258). In East Asia, the universal DW in Chinese children reaches 0.4160, compared to 0.3142 in adults, indicating that dengue infection in children can lead to more serious health consequences than in adults [72]. Policymakers should focus on prioritizing pediatric healthcare infrastructure, particularly in high-burden regions like South Asia, to address these disparities effectively. Furthermore, severe complications such as DHF and DSS significantly increase patients' DW values and are accompanied by more pronounced functional limitations [73, 74]. These complex cases often exhibit prolonged recovery periods, with patients potentially experiencing persistent symptoms including fatigue, myalgia, and cognitive difficulties for weeks or even months. This condition, termed "post-dengue fatigue syndrome," not only substantially impacts patients' quality of life and productivity but is also frequently underestimated in overall disease burden assessments, warranting greater attention in disease management strategies [75]. These findings highlight the need for age-targeted interventions and resource prioritization in dengue-endemic regions.

### Regional disparities in disability weights and data representation

The study also identifies significant regional variations in DWs for dengue. Our DW calculations across 12 countries indicate marked differences in dengue burden by region. For universal DWs, Middle Eastern countries such as Saudi Arabia showed the highest values (0.4606), followed by South Asian countries (Afghanistan: 0.3811; India: 0.3700) and Southeast Asia (Vietnam: 0.3761). Lower DWs were observed in Tanzania (0.2368) and Thailand (0.2753). In addition to English-language literature, our study extracted information from 28 Chinese-language publications, providing robust support for obtaining dengue DWs that more accurately reflect the situation in China. This comprehensive approach enhanced the precision of our estimates and reduced potential biases that might arise from language-restricted literature reviews [76]. As Bhatt et al. noted, such inclusive methodologies are essential for accurate burden estimation, particularly in regions where significant research may be published in local languages [77].

These regional variations in DWs can be attributed to multiple factors. First, the diverse distribution of dengue virus serotypes, with DENV-1 and DENV-2 primarily in Asia and the Americas, and DENV-3 and DENV-4 more common in South America, Southeast Asia, and the Caribbean, potentially influencing clinical manifestations and subsequent DWs [78]. Second, disparities in healthcare infrastructure and disease management play critical roles in regional DW values [3]. For example, South Asian countries like India, Pakistan and Afghanistan showed higher DWs, potentially due to limited resources that delay treatment and exacerbate outcomes [79]. In such regions, international collaboration to improve healthcare access and strengthen vector control programs could play a transformative role in reducing disease burden [80, 81]. In contrast, regions with strong healthcare systems, like Singapore [78, 82], have low disease burden due to timely and effective case management which reduces the severity of the disease. This contrast emphasizes the importance of equitable access to healthcare resources and infrastructure investments in resource-limited settings [83]. Finally, differences in data collection and monitoring systems can affect DW accuracy [84]. Standardized systems, such as the United States Center for Diseases Control and Prevention's National Notifiable Disease Surveillance System, report and monitor dengue cases by providing a consistent electronic health record system [85], whereas non-standardized data collection can lead to instability in DW estimates [86].

Our study outlines the DWs for South Asian regions, but limited data from Latin America restricts the reliability of estimates for this high-incidence area. Despite Brazil’s high dengue burden, few studies from Brazil met our inclusion criteria, potentially due to research priorities focused more on vector control than clinical manifestations [87]. This gap in research from high-burden regions like Brazil underscores the urgency of expanding local language studies to fill critical data gaps [88]. Expanding data collection efforts in regions like Latin America, coupled with integrating multi-language literature, would provide a more holistic understanding of global dengue burden. Moreover, future research could apply this study's methodology to other global high-burden areas, such as sub-Saharan Africa, to further refine resource allocation strategies. These regional differences underscore the need for context-specific assessments of dengue burden, accounting for each region's unique epidemiological profile, healthcare infrastructure, and public health investment.

### Strengths and limitations

This study provides a comprehensive assessment of global dengue clinical manifestations and DWs, addressing critical gaps in age- and region-specific variations overlooked by previous GBD studies. The use of Monte Carlo simulations to estimate uncertainty intervals enhances the precision and reliability of DWs, making the findings highly applicable for public health decision-making. Additionally, the inclusion of Chinese and English literature expands the scope of data sources compared to prior analyses, increasing the study's relevance for diverse populations.

However, the study has limitations. First, the exclusion of non-English and non-Chinese studies, particularly those in Spanish and Portuguese, may have underrepresented regions like Latin America. Second, the lack of differentiation by dengue serotypes (DENV-1 to DENV-4) and infection history (primary vs. secondary) restricts insights into factors influencing disease severity. Finally, variability in clinical reporting standards and study designs across included datasets may have introduced heterogeneity, highlighting the need for standardized global data collection in future research. Including these factors in future studies will contribute to a better understanding of different clinical outcomes and their impact on disease burden.

## Conclusions

Notable age-related and regional disparities in dengue clinical manifestations and disease burden were identified in this study, providing detailed insights into the disease's impact across different regions. These disparities highlight the need for localized research and tailored public health interventions. Countries with high dengue incidence, such as Brazil, Mexico, Colombia, and Paraguay, are underrepresented in global clinical manifestations research, creating critical data gaps. Improving clinical data collection in underrepresented regions is essential for optimizing dengue control efforts and ensuring more equitable resource allocation globally.

## Supplementary Information

Supplementary Material 1: MOOSE Checklist.

Supplementary Material 2: Search strategy for each database.

Supplementary Material 3: Studies included in the review and meta-analysis.

Supplementary Material 4: Quality assessment.

Supplementary Material 5: Disability Weights and Log-Normal Distribution of Dengue Clinical Manifestations, odds ratio (*OR*) and Chi-Square Test Results for Population Difference.

Supplementary Material 6: The global distribution of dengue-related YLDs across countries in 2021.

Supplementary Material 7: The global distribution of dengue-related clinical English-language literature.

## Data Availability

Relevant data are available from the corresponding author on request.

## Abbreviations

DWs: Disability weights
GBD: Global burden of disease
WHO: World Health Organization
DHF: Dengue hemorrhagic fever
DSS: Dengue shock syndrome
DALY: Disability-adjusted life year
YLL: Years of life lost
YLD: Years lived with disability
*CI*: Confidence interval
UI: Uncertainty interval
*OR*s: Odds ratios

## References

[1] Brady OJ, Gething PW, Bhatt S, Messina JP, Brownstein JS, Hoen AG, et al. Refining the global spatial limits of dengue virus transmission by evidence-based consensus. PLoS Negl Trop Dis. 2012;6(8): e1760. 22880140 10.1371/journal.pntd.0001760 PMC3413714

[2] World Health Organization. WHO results report: Region of the Americas. 2024. https://www.who.int/about/accountability/results/who-results-report-2024-2025/region-AMRO. Accessed 16 May 2025.

[3] Stanaway JD, Shepard DS, Undurraga EA, Halasa YA, Coffeng LE, Brady OJ, et al. The global burden of dengue: an analysis from the Global Burden of Disease Study 2013. Lancet Infect Dis. 2016;16(6):712–23. 26874619 10.1016/S1473-3099(16)00026-8 PMC5012511

[4] World Health Organization. Ending the neglect to attain the sustainable development goals: a road map for neglected tropical diseases 2021–2030. Geneva: World Health Organization, 2020. https://www.who.int/publications/i/item/9789240010352. Accessed 16 May 2025.

[5] Murray CJ, Lopez AD. Measuring the global burden of disease. N Engl J Med. 2013;369(5):448–57. 10.1056/NEJMra1201534. 23902484 10.1056/NEJMra1201534

[6] Anderson KB, Chunsuttiwat S, Nisalak A, Mammen MP, Libraty DH, Rothman AL, et al. Burden of symptomatic dengue infection in children at primary school in Thailand: a prospective study. Lancet. 2007;369(9571):1452–9. 10.1016/S0140-6736(07)60671-0. 17467515 10.1016/S0140-6736(07)60671-0

[7] Shepard DS, Undurraga EA, Halasa YA, Stanaway JD. Economic and disease burden of dengue in Southeast Asia. PLoS Negl Trop Dis. 2016. 10.1371/journal.pntd.0004721. 23437406 10.1371/journal.pntd.0002055 PMC3578748

[8] Stroup DF, Berlin JA, Morton SC, et al. Meta-analysis of observational studies in epidemiology: a proposal for reporting. JAMA. 2000;283(15):2008–12. 10.1001/jama.283.15.2008. 10789670 10.1001/jama.283.15.2008

[9] XueShuDianDi. COOC is a software for bibliometrics and knowledge graph drawing. https://gitee.com/academic_2088904822/academic-drip. Accessed 3 Jun 2025.

[10] World Health Organization. Dengue: guidelines for diagnosis, treatment, prevention, and control. New Edition. Geneva: World Health Organization; 2009. https://www.who.int/publications/i/item/9789241547871. Accessed 16 May 2025. 23762963

[11] World Health Organization. Dengue haemorrhagic fever: Diagnosis, treatment, prevention and control. 2nd ed. Geneva: World Health Organization; 1997. https://www.who.int/publications/i/item/9241545000. Accessed 16 May 2025.

[12] Global Health Data Exchange. GBD 2021. Institute for Health Metrics and Evaluation. https://ghdx.healthdata.org/gbd-2021. Accessed 7 Oct 2024.

[13] Global Health Data Exchange. GBD 2017. Institute for Health Metrics and Evaluation. https://ghdx.healthdata.org/gbd-2017.Accessed 7 Oct 2024.

[14] Zeng XT, Liu H, Chen X, et al. Meta-analysis series IV: quality assessment tools for observational studies. Chin J Evid Based Cardiovasc Med. 2012;4(04):297–9.

[15] Ye S, Chen Y, Xie Q. Meta-analysis of single proportions equal to 0 or 1 and its software implementation. Chin J Health Stat. 2022;39(02):316–20 (in Chinese).

[16] Feng Y, et al. Estimation of disability weight for paragonimiasis: a systematic analysis. Infect Dis Poverty. 2018;7:110. 10.1186/s40249-018-0496. 30342548 10.1186/s40249-018-0485-5 PMC6196032

[17] Qian ZX, Huang SY. Clinical analysis of 110 cases of dengue. J First Mil Med Univ. 1981;03:16–8 (in Chinese).

[18] Khan NA, Azhar EI, El-Fiky S, Madani HH, Abuljadial MA, Ashshi AM, et al. Clinical profile and outcome of hospitalized patients during first outbreak of dengue in Makkah, Saudi Arabia. Acta Trop. 2008;105(1):39–44. 10.1016/j.actatropica.2007.09.005. 17983609 10.1016/j.actatropica.2007.09.005

[19] Aung KL, Thanachartwet V, Desakorn V, Chamnanchanunt S, Sahassananda D, Chierakul W, Pitisuttithum P. Factors associated with severe clinical manifestation of dengue among adults in Thailand. Southeast Asian J Trop Med Public Health. 2013;44(4):602. 24050093

[20] Kuo HJ, Lee K, Liu JW. Analyses of clinical and laboratory characteristics of dengue adults at their hospital presentations based on the World Health Organization clinical-phase framework: emphasizing risk of severe dengue in the elderly. J Microbiol Immunol Infect. 2018;51(6):740–8. 28734676 10.1016/j.jmii.2016.08.024

[21] Taylor WR, Fox A, Pham KT, Le HNM, Tran NTH, Tran GV, et al. Dengue in adults admitted to a referral hospital in Hanoi, Vietnam. Am J Trop Med Hyg. 2015;92(6):1148–52. 10.4269/ajtmh.14-0472 PMC4458817 25918201

[22] Nazeer A, Kamal A, Qaisera S, Waheed K. Dengue fever outbreak in Lahore, Pakistan: a clinical management experience. Pak J Med Health Sci. 2009;3:204.

[23] Zheng XY. Clinical characteristics and nursing of 25 elderly dengue patients. J Mod Med Health. 2013;29(21) (in Chinese).

[24] Thanachartwet V, Oer-Areemitr N, Chamnanchanunt S, Sahassananda D, Jittmittraphap A, Suwannakudt P, et al. Identification of clinical factors associated with severe dengue among Thai adults: a prospective study. BMC Infect Dis. 2015;15:1–1. 26468084 10.1186/s12879-015-1150-2 PMC4606996

[25] Kaur G, Ahluwalia G, Singh S, Aggarwal R, Bhatia A, Malhotra P. Look out for fever: clinical profile of dengue in young adults in a tertiary care center in North India. J Lab Physicians. 2022;15(1):78–83. 37064990 10.1055/s-0042-1751320 PMC10104709

[26] Temprasertrudee S, Thanachartwet V, Desakorn V, Keatkla J, Chantratita W, Kiertiburanakul S. A multicenter study of clinical presentations and predictive factors for severe manifestation of dengue in adults. Jpn J Infect Dis. 2018;71(3):239–43. 29709965 10.7883/yoken.JJID.2017.457

[27] Liang SQ, Wu XL, Zhao MC, Ding PP, Wu HM. Clinical diagnosis and treatment characteristics of 68 dengue patients. J Trop Dis Parasitol. 2014;12(2) (in Chinese).

[28] Boillat-Blanco N, Klaassen B, Mbarack Z, Samaka J, Mlaganile T, Masimba J, et al. Dengue fever in Dar es Salaam, Tanzania: clinical features and outcome in populations of black and non-black racial category. BMC Infect Dis. 2018;18(1):644. 10.1186/s12879-018-3549-z. 30541456 10.1186/s12879-018-3549-z PMC6292068

[29] Li Y, He AH. Clinical characteristics analysis of 199 dengue patients. Int Med Health Guid News. 2019;25(8):1242–4. 10.3760/cma.j.issn.1007-1245.2019.08.018. (in Chinese).

[30] Wu HM, Bao XJ, Li Q, Yang L, Ma QZ. Epidemiological characteristics of a dengue outbreak at a university in Guangzhou in 2014. China School Health. 2016;37(04):627–9. 10.16835/j.cnki.1000-9817.2016.04.046. (in Chinese).

[31] Song FQ, Jiang LY, Huang Y, Fang XS, Yu T, Zhou LL, et al. Clinical analysis of 89 dengue cases in 2014. Chin J Hyg Rescue. 2015;1:35–7. 10.3877/cma.j.issn.2095-9133.2015.01.010. (in Chinese).

[32] Lin YP, Luo Y, Chen Y, Lamers MM, Zhou Q, Yang XH, et al. Clinical and epidemiological features of the 2014 large-scale dengue outbreak in Guangzhou city, China. BMC Infect Dis. 2016;16:102. 10.1186/s12879-016-1379-4. 26932451 10.1186/s12879-016-1379-4 PMC4774186

[33] Padyana M, Karanth S, Vaidya S, Gopaldas JA. Clinical profile and outcome of dengue fever in multidisciplinary intensive care unit of a tertiary level hospital in India. Indian J Crit Care Med. 2019;23(6):270. 31435145 10.5005/jp-journals-10071-23178 PMC6698353

[34] Zhou D, Hong YJ, Sun MF, Lin YX, Lin DF, Lin K. Analysis of factors influencing the course of dengue and its clinical characteristics. J Shantou Univ Med Coll. 2019;32(3):154–6 (in Chinese).

[35] Bisoyi SK, Behera TR, Patnaik N, Pradhan A. Clinical profile of dengue fever at SCB Medical College and Hospital, Cuttack, Odisha. J Commun Dis. 2018;50(4):1–6.

[36] Yu J, Bao XJ, Wang H, Dong M. Clinical characteristics and nursing analysis of 34 cases of dengue. Massage Rehabil Med. 2017;8(20):88–9, 91. 10.19787/j.issn.1008-1879.2017.20.044. (in Chinese).

[37] Ren H, Li X, Zhou J, Jiang F, Huang S, Lin Q. A survey of clinical and laboratory characteristics of the dengue fever epidemic from 2017 to 2019 in Zhejiang, China. Medicine. 2022. 10.1097/MD.0000000000031143. 36281095 10.1097/MD.0000000000031143 PMC9592481

[38] Pereira MS, Kudru CU, Nair S, Thunga G, Kunhikatta V, Guddattu VA. Factors associated with severity of illness in patients with dengue fever in a tertiary care hospital in southern India. Asian J Pharm Clin Res. 2018;11(3):272–6.

[39] Sediqi MF, Azimi F, Sayed S, Hashimi SM, Salehi O, Qaderi SM. Dengue fever as an emerging disease in Afghanistan: epidemiology of the first reported cases. Int J Infect Dis. 2021;104:624–9. 10.1016/j.ijid.2021.02.045. 32738489 10.1016/j.ijid.2020.07.033

[40] Chen QB, Chen MX, Lin ZD. Epidemiological and clinical characteristics analysis of dengue in Anxi County in 2019. Strait J Prev Med. 2020;26(6) (in Chinese).

[41] Rafi A, Mousumi AN, Ahmed R, Chowdhury RH, Wadood A, Hossain G. Dengue epidemic in a non-endemic zone of Bangladesh: clinical and laboratory profiles of patients. PLoS Negl Trop Dis. 2020;14(10): e0008567. 33048921 10.1371/journal.pntd.0008567 PMC7553334

[42] Ren HQ, Zhong S, Zhou Z. Epidemiological characteristics and clinical features of dengue in Chongqing in 2019. Chin Sci Technol J Database Med. 2021;(11) (in Chinese).

[43] Deng L, Hu Z, Wang J, Chen ST. Clinical characteristics analysis of 201 adult dengue cases in Liangjiang New Area, Chongqing. J Chongqing Med Univ. 2022;47(09):1111–5. 10.13406/j.cnki.cyxb.002664. (in Chinese).

[44] Liu FW, Xu SS, Yang YD. Clinical data analysis of 82 cases of dengue. Shandong Med J. 2023;63(26):65–8. 10.3969/j.issn.1002-266X.2023.26.016. (in Chinese).

[45] Liao LY, Liu QF. Clinical analysis of 90 cases of dengue in children. New Med. 1997;28(3) (in Chinese).

[46] Chen KQ. Clinical analysis of 659 cases of dengue in children. J Clin Pediatr. 1989;7(4):205–6 (in Chinese).

[47] Yang ZY, Huang YZ. Clinical analysis of 331 cases of dengue in children. Hainan Med J. 1986;3:32–4 (in Chinese).

[48] Yang J, Mei FS. Clinical analysis of 70 cases of dengue in children. Nat Sci Hainan Univ. 1993;11(3):76–8 (in Chinese).

[49] Huang JZ, Chen Z. Clinical analysis of 210 cases of dengue in children. Hainan Med J. 1989;03:11–4 (in Chinese).

[50] Feng LX. Clinical analysis of 454 cases of dengue in children. J Guangdong Med Univ. 1991;03:183–4 (in Chinese).

[51] Yu TT, Luo F, Liu JX, Guo HX, Ma YL, Luo X, et al. Clinical characteristics of pediatric dengue cases during the 2014 dengue outbreak in Guangzhou. China Trop Med. 2016;16(03):273–6. 10.13604/j.cnki.46-1064/r.2016.03.20. (in Chinese).

[52] Fang CX, Xu Y, Tan LM, Ye JW, Luo D, Yang FX, et al. Clinical analysis of 78 pediatric dengue cases. Chin J Infect Dis. 2018;36(9) (in Chinese).

[53] Jia SJ, Liu W, Tan XH, Lin LP, Fan HM, Xie M. Clinical characteristics and laboratory examination analysis of pediatric dengue patients in Guangzhou. J Trop Med. 2019;0(10) (in Chinese).

[54] Lin J, Hong WX, Zhang FC, Hu D, Tan LL. Clinical characteristics analysis of 34 cases of dengue in infants and young children. J Pediatr Pharmacol. 2016;22(7) (in Chinese).

[55] Guo JQ, Sun GY, Li CH, et al. Clinical analysis of 33 pediatric dengue cases. Guangdong Med J. 2016;37(z1):153–4 (in Chinese).

[56] Jayarajah U, Madarasinghe M, Hapugoda D, Dissanayake U, Perera L, Kannangara V, et al. Clinical and biochemical characteristics of dengue infections in children from Sri Lanka. Glob Pediatr Health. 2020;7:2333794X20921524. 10.1177/2333794X20921524. 10.1177/2333794X20974207 PMC7686613 33283028

[57] Lue AM, Richards-Dawson MA, Gordon-Strachan GM, Kodilinye SM, Dunkley-Thompson JA, James-Powell TD, et al. Severity and outcomes of Dengue in hospitalized Jamaican children in 2018–2019 during an epidemic surge in the Americas. Front Med. 2022;9: 889998. 10.3389/fmed.2022.889998 PMC9254731 35801209

[58] Wu XM, Deng YY, Gan Y, Yang D. Clinical analysis of dengue fever in children. Chin Sci Tech J Database Med Health. 2021;2:104–5 (in Chinese).

[59] Hong WX, Wang J, Qiu S, Li YP, Yang HQ, Tan XH, et al. Clinical characteristics and treatment experience of 121 adult cases with severe dengue fever. J Sun Yat-sen Univ. 2016;37(3):333–6 (in Chinese).

[60] Wu H, Lu YH, Xu ZB. Clinical analysis of 65 severe dengue fever cases in Guangzhou. Med Front. 2014;27:204–5. 10.3969/j.issn.2095-1752.2014.27.194. (in Chinese).

[61] Hegazi MA, Bakarman MA, Alahmadi TS, Butt NS, Alqahtani AM, Aljedaani BS, et al. Risk factors and predictors of severe dengue in Saudi population in Jeddah, Western Saudi Arabia: a retrospective study. Am J Trop Med Hyg. 2020;102(3):613. 31933467 10.4269/ajtmh.19-0650 PMC7056408

[62] Li XP, Ye QX. Nursing care of 16 patients with severe dengue fever. Mod Clin Nurs. 2009;8(11):38–40 (in Chinese).

[63] He HL, Zhang FC, Chen YQ, Wang J. Clinical analysis of 36 cases of severe dengue fever in Guangzhou. Trop Dis Parasitol. 2005;3(2):89–91 (in Chinese).

[64] Wichmann O, Hongsiriwon S, Bowonwatanuwong C, Chotivanich K, Sukthana Y, Pukrittayakamee S. Risk factors and clinical features associated with severe dengue infection in adults and children during the 2001 epidemic in Chonburi, Thailand. Trop Med Int Health. 2004;9(9):1022–9. 15361117 10.1111/j.1365-3156.2004.01295.x

[65] Bhattacharya D, Angurana SK, Nallasamy K, Iyer R, Jayashree M. Severe dengue and associated hemophagocytic lymphohistiocytosis in PICU. Indian Pediatr. 2019;86:1094–8. 10.1007/s12098-019-03040-0 31353429

[66] Nguyen RN, Lam HT, Phan HV, Lam HT, Phan HV. Liver impairment and elevated aminotransferase levels predict severe dengue in Vietnamese children. Cureus. 2023;15(10): e47606. 37886653 10.7759/cureus.47606 PMC10597804

[67] Branco MD, Luna EJ, Braga LL, Oliveira RV, Rios LT, Silva MD, et al. Risk factors associated with death in Brazilian children with severe dengue: a case-control study. Clinics. 2014;69(1):55–60. 24473560 10.6061/clinics/2014(01)08 PMC3870309

[68] Wu XM, Deng YY, Gan Y, Yang D. Clinical analysis of dengue in children. Med Health. 2021;2:104–5 (in Chinese).

[69] Ouyang H, Zhao Y, Hong L, et al. Changing trends of dengue in China: Meta-analysis of comorbidity rates and clinical manifestations. China Trop Med. 2024;24(8):900–7. 10.13604/j.cnki.46-1064/r.2024.08.03. (in Chinese).

[70] World Health Organization. Dengue: Guidelines for Diagnosis, Treatment, Prevention and Control. World Health Organization; 2012. https://www.who.int/publications/i/item/9789241504034. Accessed 16 May 2025.

[71] Halstead SB. Dengue in the Americas and Southeast Asia: do they differ. Rev Panam Salud Publica. 2003;14(4):217–22. 10.1590/s1020-49892003000900001. 10.1590/s1020-49892006001100007 17341332

[72] Guzman MG, Gubler DJ, Izquierdo A, et al. Dengue: a continuing global threat. Nat Rev Microbiol. 2010. 10.1038/nrmicro2460. 21079655 10.1038/nrmicro2460 PMC4333201

[73] World Health Organization. Dengue hemorrhagic fever: a global update. World Health Organization. 2020. https://www.who.int/denguecontrol/update/en/. Accessed 16 May 2025.

[74] Dung NM, Thoang DD, Phuong HT, et al. Severe dengue in Vietnam: an overview of clinical features and management of complications. Southeast Asian J Trop Med Public Health. 2019;50(4):655–62.

[75] Tian Y, Cazelles B, Simmons C, Lau CL. Post-dengue chronic fatigue syndrome: a systematic review and meta-analysis of cohort studies. J Infect Dis. 2022;225(9):1574–86.

[76] Liu W, Huang B, Zhan J, Wu J, Zhang Y, Zhou G, et al. Meta-analysis of dengue fever studies from China: revealing new challenges and opportunities. PLoS Negl Trop Dis. 2020;14(5).

[77] Bhatt S, Gething PW, Brady OJ, Messina JP, Farlow AW, Moyes CL, et al. The global distribution and burden of dengue. Nature. 2013;496(7446):504–7. 23563266 10.1038/nature12060 PMC3651993

[78] Shepard DS, Undurraga EA, Halasa YA, Stanaway JD. The global economic burden of dengue: a systematic analysis. Lancet Infect Dis. 2016;16(8):935–41. 27091092 10.1016/S1473-3099(16)00146-8

[79] Messina JP, Brady OJ, Scott TW, Zou C, Pigott DM, Duda KA, et al. Global spread of dengue virus types: mapping the 70-year history. Trends Microbiol. 2014;22(3):138–46. 24468533 10.1016/j.tim.2013.12.011 PMC3946041

[80] Wilder-Smith A, Tissera H, AbuBakar S, Kittayapong P, Logan J, Neumayr A, et al. Novel tools for the surveillance and control of dengue: findings by the DengueTools research consortium. Glob Health Action. 2018;11(1):1549930. 30560735 10.1080/16549716.2018.1549930 PMC6282436

[81] Achee NL, Gould F, Perkins TA, Reiner RC Jr, Morrison AC, Ritchie SA, et al. A critical assessment of vector control for dengue prevention. PLoS Negl Trop Dis. 2015;9(5): e0003655. 25951103 10.1371/journal.pntd.0003655 PMC4423954

[82] Lee LK, Thein TL, Gan VC, Lye DC, Leo YS. Dengue knowledge, attitudes, and practices among primary care physicians in Singapore. Ann Acad Med Singap. 2011;40(12):533–8. 22294064

[83] Quintero J, Brochero H, Manrique-Saide P, Barrera-Pérez M, Basso C, Romero S, et al. Ecological, biological and social dimensions of dengue vector breeding in five urban settings of Latin America: a multi-country study. BMC Infect Dis. 2014;14:38. 24447796 10.1186/1471-2334-14-38 PMC3904013

[84] Vong S, Goyet S, Ly S, Ngan C, Huy R, Duong V, et al. Under-recognition and reporting of dengue in Cambodia: a capture-recapture analysis of the National Dengue Surveillance System. Epidemiol Infect. 2012;140(3):491–9. 21733251 10.1017/S0950268811001191

[85] World Economic Forum. Dengue: integrated management to achieve zero deaths. 2023. https://www.weforum.org/agenda/2023/11/dengue-integrated-management-to-achieve-zero-deaths/. Accessed 28 Oct 2024.

[86] World Health Organization. Global strategy for dengue prevention and control 2012–2020. Geneva: World Health Organization; 2012. https://www.who.int/denguecontrol/strategy/en/. Accessed 16 May 2025.

[87] Teixeira MG, et al. Epidemiological trends of dengue disease in Brazil (2000–2010): a systematic literature review. PLoS Negl Trop Dis. 2013. 10.1371/journal.pntd.0002520. 24386496 10.1371/journal.pntd.0002520 PMC3871634

[88] Oliveira RMAB, Araújo FMC, Cavalcanti LPG. Challenges for performing clinical research on dengue in Brazil. Rev Soc Bras Med Trop. 2018;51(5):683–5.30304279

