# Supplementary material for: Global estimation of dengue disability weights based on clinical manifestations data
Source: Infect Dis Poverty. 2025 Jun 9;14:44. doi: 10.1186/s40249-025-01317-5 (PMC12147332; doi:10.1186/s40249-025-01317-5)
Supplement: Supplementary file 1 — Supplementary Material 1: MOOSE Checklist. [file 40249_2025_1317_MOESM1_ESM.docx]

**Supplementary file 1.** MOOSE Checklist

A Proposed Reporting Checklist for Authors, Editors, and Reviewers of Meta-analyses of Observational Studies

| **Reporting of background should include** | **Description** | **Page** |
| --- | --- | --- |
| Problem Definition | The article identifies the need to assess age and region-specific differences in clinical manifestations and disability weights (DWs)associated with dengue globally. | Page 4 |
| Type of Study Designs Used | Systematic review and meta-analysis of observational studies on dengue clinical data | Page 5 |
| Study Population | Included studies focus on human populations affected by dengue, divided into adult and pediatric groups. | Page 4-5 |
| **Reporting of Search Strategy Should Include** | **Description** | **Page** |
| Qualifications of Searchers | Search conducted independently by three authors. | Page 7 |
| Search Strategy (including time and keywords) | Comprehensive search in six databases using terms such as (dengue OR dengue fever OR dengue hemorrhagic fever OR breakbone fever OR bouquet fever OR chapenonada OR dengue shock syndrome) AND ("clinical" OR "infection" OR “manifestation”). | Page 6 |
| Effort to Include All Available Studies (including contact with authors) | Contact with authors as necessary for additional data and clarification. | Supplementary file |
| Databases and Registries Searched | Scopus, Web of Science, PubMed, CNKI, Wanfang, and VIP. | Page 5-6 |
| Search Software, Name, Version, Features | EndNote and Excel for record management; COOC 13.9 for deduplication. | Page 6 |
| Use of Hand Searching | Not specified, but reference lists of included studies were reviewed for additional sources. | Page 5-7 |
| List of Citations Located and Excluded (including justification) | Details provided in the study flowchart (Figure 1). | Figure 1 |
| Method of Addressing Articles in Languages Other Than English | Included English and Chinese articles only. | Page 6 |
| Method of Handling Abstracts and Unpublished Studies | Excluded studies without full text or complete data. | Page 6 |
| **Reporting of Methods Should Include** | **Description** | **Page** |
| Rationale for Selection and Coding of Data | Consistency with WHO guidelines and clinical relevance. | Page 6 |
| Documentation of Data Classification and Coding (e.g., multiple raters) | Data independently extracted by three authors with resolution of discrepancies through discussion. | Page 6 |
| Assessment of Study Quality (including blinding of quality assessors) | Studies assessed for data completeness and accuracy; blinding not applicable due to open data sources. | Page 6-8 |
| Assessment of Heterogeneity | Heterogeneity evaluated using Q-test and I² statistic. | Page 8 |
| Description of Statistical Methods | Random-effects model for meta-analysis; Chi-square tests; Monte Carlo simulation for uncertainty intervals. | Page 8-9 |
| Provision of Appropriate Tables and Graphics | Incidence rates and DWs visualized in figures | Figures 1-3 and Table 1 |
| **Reporting of Results Should Include** | **Description** | **Page** |
| Graphic Summaries of Individual Study Estimates and Overall Estimates | Visualized through forest plots and bar charts | Figures 2-3 |
| Tables Giving Descriptive Information | Detailed tables of clinical manifestations, DWs, and severity by age and region. | Table 1 and Supplementary file 1-7 |
| Results of Sensitivity Testing | Sensitivity tested via random-effects model and Monte Carlo simulations. | Page 10, 11 |
| Indication of Statistical Uncertainty of Findings | Uncertainty intervals provided in the results. | Page 11 |
| **Reporting of Discussion Should Include** | **Description** | **Page** |
| Justification of Exclusion | Exclusions justified based on relevance and data completeness. | Page 14-18 |
| Assessment of Quality of Included Studies | Quality assessed based on data availability and accuracy criteria. | Page 14-18 |
| **Reporting of Conclusions Should Include** | **Description** | **Page** |
| Consideration of Alternative Explanations for Observed Results | Possible causes for regional and age-specific differences discussed. | Page 14-16 |
| Generalization of Conclusions | Conclusions applicable to global dengue management strategies. | Page 17-18 |
| Guidelines for Future Research | Suggestions for further studies in underrepresented regions like Latin America. | Page 18-19 |
| Disclosure of Funding Source | Funded by the National Natural Science Foundation of China and other sources. | Page 20-21 |
