# Supplementary material for: Global estimation of dengue disability weights based on clinical manifestations data
Source: Infect Dis Poverty. 2025 Jun 9;14:44. doi: 10.1186/s40249-025-01317-5 (PMC12147332; doi:10.1186/s40249-025-01317-5)
Supplement: Supplementary file 2 — Supplementary Material 2: Search strategy for each database. [file 40249_2025_1317_MOESM2_ESM.docx]

**Supplementary file 2.** Search strategy for each database

For English databases Scopus: (TITLE-ABS-KEY (dengue) OR TITLE-ABS-KEY (dengue AND fever) OR TITLE-ABS-KEY (dengue AND hemorrhagic AND fever) OR TITLE-ABS-KEY (breakbone AND fever) OR TITLE-ABS-KEY (bouquet AND fever) OR TITLE-ABS-KEY (chapenonada) OR TITLE-ABS-KEY (dengue AND shock AND syndrome)) AND PUBYEAR < 2024 AND (TITLE-ABS-KEY(clinical) OR TITLE-ABS-KEY (infection) OR TITLE-ABS-KEY (manifestation)) AND (LIMIT-TO(SUBJAREA, "MEDI")) AND (LIMIT-TO(DOCTYPE, "ar")) AND (LIMIT-TO(LANGUAGE , "English"))

For English databases Web of Science: dengue OR dengue fever OR dengue hemorrhagic fever OR breakbone fever OR bouquet fever OR chapenonada OR dengue shock syndrome (All Fields) and clinical OR infection OR manifestation (All Fields) Publication Year: -2023, Language: English

For English databases PubMed: (clinical OR infection OR manifestation) AND (dengue OR dengue fever OR dengue hemorrhagic fever OR breakbone fever OR bouquet fever OR chapenonada OR dengue shock syndrome) Filters: Free full text, English, Humans, from 1950 - 2023

For Chinese databases such as China National Knowledge Infrastructure, Wanfang Data, and Database of Chinese Sci-Tech Periodicals, the search terms used were translated as follows: (Topic: (Dengue OR Dengue Fever OR Dengue Hemorrhagic Fever OR Breakbone Fever OR Dengue Shock Syndrome) AND Topic: (Infection OR Clinical OR Manifestation)) AND Publication Date: *-2023 AND Access Range: Full Text Available AND Language: Chinese
