# Supplementary material for: Global estimation of dengue disability weights based on clinical manifestations data
Source: Infect Dis Poverty. 2025 Jun 9;14:44. doi: 10.1186/s40249-025-01317-5 (PMC12147332; doi:10.1186/s40249-025-01317-5)
Supplement: Supplementary file 3 — Supplementary Material 3: Studies included in the review and meta-analysis. [file 40249_2025_1317_MOESM3_ESM.docx]

**Supplementary file 3**. Studies included in the review and meta-analysis

| Number | Title | Authors | Year | DOI | Study population | Country | Age range | Survey time | Total number of cases | Number of dengue cases | Number of severe dengue cases |
| --- | --- | --- | --- | --- | --- | --- | --- | --- | --- | --- | --- |
| 1 | Clinical Analysis of 110 Cases of Dengue Fever (in Chinese) | Qian ZX et al | 1981 | - | Adult | China | 15-50year | 1980.08-1980.10 | 110 | 110 | 0 |
| 2 | Clinical profile and outcome of hospitalized patients during first outbreak of dengue in Makkah, Saudi Arabia | Nasim A. Khan et al | 2008 | [10.1016/j.actatropica.2007.09.005](https://doi.org/10.1016/j.actatropica.2007.09.005) | Adult | Saudi Arabia | 34-90.6year | 2004.04-2004.07 | 91 | 85 | 6 |
| 3 | Factors associated with severe clinical manifestation of dengue among adults in Thailand | Aung KLL et al | 2013 | - | Adult | Thailand | ≥15year | 2006.01-2010.12 | 323 | 233 | 90 |
| 4 | Analyses of clinical and laboratory characteristics of dengue adults at their hospital presentations based on the World Health Organization clinical-phase framework: Emphasizing risk of severe dengue in the elderly | Kuo HJ et al | 2018 | [10.1016/j.jmii.2016.08.024](https://doi.org/10.1016/j.jmii.2016.08.024) | Adult | China | ＞18year | 2008.01-2014.12 | 669 | 642 | 27 |
| 5 | Dengue in adults admitted to a referral hospital in Hanoi, Vietnam | Walter R Taylor et al | 2015 | [10.4269/ajtmh.14-0472](https://doi.org/10.4269/ajtmh.14-0472) | Adult | Vietnam | 16-72year | 2008.01-2008.12 | 143 | 139 | 4 |
| 6 | Dengue fever out break in Lahore, Pakistan. A clinical management experience | ASMA NAZEER et al | 2009 | - | Adult | Pakistan | 16-80year | 2008.09-2008.11 | 254 | 252 | 2 |
| 7 | Clinical Characteristics and Nursing Care of 25 Elderly Patients with Dengue Fever (in Chinese) | Zheng XY et al | 2013 | [10.3969/j.issn.1009-5519.2013.21.061](http://dx.chinadoi.cn/10.3969/j.issn.1009-5519.2013.21.061) | Adult | China | 60-80year | 2012.09-2012.12 | 25 | 25 | 0 |
| 8 | Identification of clinical factors associated with severe dengue among Thai adults: a prospective study | Thanachartwet V et al | 2015 | [10.1186/s12879-015-1150-2](https://doi.org/10.1186/s12879-015-1150-2) | Adult | Thailand | ≥15year | 2012.10-2014.12 | 153 | 132 | 21 |
| 9 | Look Out For Fever: Clinical Profile Of Dengue In Young Adults In A Tertiary Care Center In North India | Gursheen Kaur et al | 2023 | [10.1055/s-0042-1751320](https://doi.org/10.1055/s-0042-1751320) | Adult | India | 18-30year | 2013.01-2014.12 | 418 | 365 | 53 |
| 10 | A Multicenter Study of Clinical Presentations and Predictive Factors for Severe Manifestation of Dengue in Adults | Temprasertrudee S et al | 2018 | [10.7883/yoken.JJID.2017.457](https://doi.org/10.7883/yoken.jjid.2017.457) | Adult | Thailand | ≥15year | 2013.01-2015.07 | 357 | 319 | 38 |
| 11 | Analysis of Clinical Diagnosis and Treatment Characteristics of 68 Patients with Dengue Fever (in Chinese) | Wu HM et al | 2014 | [10.3969/j.issn.1672-2302.2014.02.010](http://dx.chinadoi.cn/10.3969/j.issn.1672-2302.2014.02.010) | Adult | China | 16-72year | 2013.08-2013.11 | 68 | 67 | 1 |
| 12 | Dengue fever in Dar es Salaam, Tanzania: Clinical features and outcome in populations of black and non-black racial category | Noémie Boillat-Blanco et al | 2018 | [10.1186/s12879-018-3549-z](https://doi.org/10.1186/s12879-018-3549-z) | Adult | Tanzania | ＞18year | 2013.12-2014.07 | 428 | 408 | 20 |
| 13 | Analysis of Clinical Characteristics of 199 Dengue Fever Patients (in Chinese) | He AH et al | 2019 | [10.3760/cma.j.issn.1007-1245.2019.08.018](http://dx.chinadoi.cn/10.3760/cma.j.issn.1007-1245.2019.08.018) | Adult | China | 17-94year | 2014.06-2014.12 | 199 | 199 | 0 |
| 14 | The epidemic characteristics of a dengue fever outbreak in a certain university in Guangzhou in 2014 (in Chinese) | YangLing et al | 2016 | 10.16835/j.cnki.1000-9817.2016.04.046 | Adult | China | 18-21year | 2014.09-2014.10 | 34 | 34 | 0 |
| 15 | Clinical Analysis of 89 Cases of Dengue Fever in 2014 (in Chinese) | YuTao et al | 2015 | - | Adult | China | 19-88year | 2014.08-2014.11 | 89 | 89 | 0 |
| 16 | Clinical and epidemiological features of the 2014 large-scale dengue outbreak in Guangzhou city, China | Yong Ping Lin et al | 2016 | [10.1186/s12879-016-1379-4](https://doi.org/10.1186/s12879-016-1379-4) | Adult | China | 34-90.6year | 2014.09-2014.11 | 138 | 130 | 8 |
| 17 | Clinical profile and outcome of dengue fever in multidisciplinary intensive care unit of a tertiary level hospital in india | Padyana M et al | 2019 | [10.5005/jp-journals-10071-23178](https://doi.org/10.5005/jp-journals-10071-23178) | Adult | India | 21-40year | 2015.07-2015.12 | 96 | 55 | 41 |
| 18 | Analysis of Influencing Factors and Clinical Characteristics of Dengue Fever Course (in Chinese) | Ling YH et al | 2019 | 10.13401/j.cnki.jsumc.2019.03.007 | Adult | China | 20-71year | 2015.08-2015.11 | 766 | 757 | 9 |
| 19 | Clinical profile of dengue fever at SCB Medical College and Hospital, Cuttack, Odisha | Sasmita Kumari Bisoyi et al | 2018 | - | Adult | India | 15-88year | 2016.07-2016.09 | 720 | 594 | 126 |
| 20 | Clinical Characteristics and Nursing Analysis of 34 Cases of Dengue Fever (in Chinese) | Bao XJ et al | 2017 | [10.3969/j.issn.1008-1879.2017.20.044](http://dx.chinadoi.cn/10.3969/j.issn.1008-1879.2017.20.044) | Adult | China | 18-45year | 2016.09-2016.11 | 34 | 34 | 0 |
| 21 | A survey of clinical and laboratory characteristics of the dengue fever epidemic from 2017 to 2019 in Zhejiang, China | Ren Ze-Ze et al | 2022 | [10.1097/MD.0000000000031143](https://doi.org/10.1097/md.0000000000031143) | Adult | China | 16-91year | 2017.08-2019.12 | 231 | 229 | 2 |
| 22 | Factors associated with severity of illness in patients with dengue fever in a tertiary care hospital in southern India | Pereira MS et al | 2018 | [10.22159/ajpcr.2018.v11i3.23496](http://dx.doi.org/10.22159/ajpcr.2018.v11i3.23496) | Adult | India | ＞18year | Not reported | 550 | 449 | 101 |
| 23 | Dengue fever as an emerging disease in Afghanistan: Epidemiology of the first reported cases | Mohammad Nadir Sahak et al | 2020 | [10.1016/j.ijid.2020.07.033](https://doi.org/10.1016/j.ijid.2020.07.033) | Adult | Afghanistan | 15-55year | 2019.01-2019.12 | 15 | 14 | 1 |
| 24 | Analysis of Epidemiological and Clinical Characteristics of Dengue Fever in Anxi County in 2019 (in Chinese) | Chen QB et al | 2020 | - | Adult | China | 16-59yaer | 2019.06-2019.10 | 65 | 65 | 0 |
| 25 | Dengue epidemic in a non-endemic zone of Bangladesh: Clinical and laboratory profiles of patients | Rafi A et al | 2020 | [10.1371/journal.pntd.0008567](https://doi.org/10.1371/journal.pntd.0008567) | Adult | Bangladesh | ＞18year | 2019.07-2019.09 | 319 | 300 | 19 |
| 26 | The Epidemiological Characteristics and Clinical Manifestations of Dengue Fever Epidemic in Chongqing City in 2019 (in Chinese) | ZhouZhi et al | 2021 | - | Adult | China | 16-69year | 2019.08-2019.10 | 36 | 36 | 0 |
| 27 | Analysis of Clinical Characteristics of 201 Adult Dengue Fever Cases in Chongqing Liangjiang New Area (in Chinese) | Chen ST et al | 2022 | 10.13406/j.cnki.cyxb.002664 | Adult | China | 16-91year | 2019.08-2019.10 | 201 | 200 | 1 |
| 28 | Analysis of Clinical Data of 82 Patients with Dengue Fever (in Chinese) | Liu FW et al | 2023 | [10.3969/j.issn.1002-266X.2023.26.016](http://dx.chinadoi.cn/10.3969/j.issn.1002-266X.2023.26.016) | Adult | China | 26-59year | 2023.08-2023.09 | 82 | 75 | 7 |
| 29 | Clinical Analysis of 90 Cases of Dengue Fever in Children (in Chinese) | Liao LY et al | 1997 | - | Child | China | ＜14year | 1980.01-1995.12 | 90 | 90 | 0 |
| 30 | Clinical Analysis of 659 Cases of Dengue Fever in Children (in Chinese) | Chen KQ et al | 1989 | - | Child | China | 3m-14year | 1980.03-1986.12 | 659 | 412 | 247 |
| 31 | Clinical Analysis of 331 Cases of Dengue Fever in Children (in Chinese) | Huang YZ et al | 1986 | - | Child | China | ＜14year | 1986.07-1986.10 | 331 | 295 | 36 |
| 32 | Clinical Analysis of 70 Cases of Dengue Fever in Children (in Chinese) | Mei FS et al | 1993 | 10.15886/j.cnki.hdxbzkb.1993.03.016 | Child | China | ＜15year | 1986.07-1986.12 | 70 | 38 | 32 |
| 33 | Clinical Analysis of 210 Cases of Dengue Fever in Children (in Chinese) | ChenChi et al | 1989 | - | Child | China | ＜15year | 1987.01-1987.12 | 210 | 132 | 78 |
| 34 | Clinical Analysis of 454 Cases of Dengue Fever in Children (in Chinese) | Feng LX et al | 1991 | - | Child | China | ＜14year | 1989.01-1989.12 | 454 | 454 | 0 |
| 35 | Clinical Characteristics of Dengue Fever Cases in Children during the 2014 Dengue Outbreak in Guangzhou City (in Chinese) | Yu TT et al | 2016 | 10.13604/j.cnki.46-1064/r.2016.03.20 | Child | China | 1-13year | 2014.08-2014.10 | 31 | 31 | 0 |
| 36 | Clinical Analysis of 78 Cases of Dengue Fever in Children (in Chinese) | Wang YL et al | 2018 | [10.3760/cma.j.issn.1000-6680.2018.09.008](http://dx.chinadoi.cn/10.3760/cma.j.issn.1000-6680.2018.09.008) | Child | China | 27d-14year | 2014.08-2014.12 | 78 | 71 | 7 |
| 37 | Analysis of Clinical Characteristics and Laboratory Tests of Dengue Fever Patients among Children in Guangzhou City (in Chinese) | LiuWei et al | 2019 | [10.3969/j.issn.1672-3619.2019.10.005](http://dx.chinadoi.cn/10.3969/j.issn.1672-3619.2019.10.005) | Child | China | 1-14year | 2014.08-2018.10 | 179 | 179 | 0 |
| 38 | Analysis of Clinical Characteristics of 34 Cases of Dengue Fever in Infants and Young Children (in Chinese) | HuDan et al | 2016 | 10.13407/j.cnki.jpp.1672-108X.2016.07.004 | Child | China | 24d-3year | 2014.09-2014.12 | 34 | 34 | 0 |
| 39 | Clinical Analysis of 33 Cases of Dengue Fever in Children (in Chinese) | Cai XP et al | 2016 | - | Child | China | 39d-12year | 2015.09-2015.11 | 33 | 33 | 0 |
| 40 | Clinical and Biochemical Characteristics of Dengue Infections in Children From Sri Lanka | U Jayarajah et al | 2020 | [10.1177/2333794X20974207](https://doi.org/10.1177/2333794x20974207) | Child | Sri Lanka | 8.6±3.3year | 2017.06-2017.08 | 305 | 245 | 60 |
| 41 | Severity and Outcomes of Dengue in Hospitalized Jamaican Children in 2018-2019 During an Epidemic Surge in the Americas | AM Lue et al | 2022 | [10.3389/fmed.2022.889998](https://doi.org/10.3389/fmed.2022.889998) | Child | Jamaica | ＜15year | 2018.08-2019.09 | 339 | 271 | 68 |
| 42 | Clinical Analysis of Dengue Fever in Children (in Chinese) | GanYun et al | 2021 | - | Child | China | 10m-14year | 2019.08-2019.12 | 15 | 15 | 0 |
| 43 | Risk factors and clinical features associated with severe dengue infection in adults and children during the 2001 epidemic in Chonburi, Thailand (in Chinese) | Ole Wichmann et al | 2016 | 10.13471/j.cnki.j.sun.yat-sen.univ(med.sci).2016.0059 | Adult | China | 15-83year | 2014.06-2014.12 | 121 | 0 | 121 |
| 44 | Identification of clinical factors associated with severe dengue among Thai adults: A prospective study (in Chinese) | Vipa Thanachartwet et al | 2014 | 10.3969/j.issn.2095-1752.2014.27.194 | Adult | China | 18-65year | 2014.04-2014.08 | 65 | 0 | 65 |
| 45 | Risk Factors and Predictors of Severe Dengue in Saudi Population in Jeddah, Western Saudi Arabia: A Retrospective Study. | Moustafa A. Hegazi et al | 2015 | [10.1186/s12879-015-1150-2](https://doi.org/10.1186/s12879-015-1150-2) | Adult | Thailand | ≥15year | 2012.10-2014.12 | 21 | 0 | 21 |
| 46 | Clinical Characteristics and Therapeutic Experience of 121 Adult Patients with Severe Dengue Fever | Cai WP et al | 2020 | [10.4269/ajtmh.19-0650](https://doi.org/10.4269/ajtmh.19-0650) | Adult | Saudi Arabia | ＞18year | 2010.01-2016.12 | 187 | 0 | 187 |
| 47 | Clinical Analysis of 65 Severe Dengue Fever Cases in Guangzhou City (in Chinese) | Lu YH et al | 2009 | [10.3969/j.issn.1671-8283.2009.11.017](http://dx.chinadoi.cn/10.3969/j.issn.1671-8283.2009.11.017) | Adult | China | 35-73year | 2007.05-2007.11 | 16 | 0 | 16 |
| 48 | Nursing care of 16 patients with severe dengue fever (in Chinese) | Ye QX et al | 2005 | [10.3969/j.issn.1672-2302.2005.02.008](http://dx.chinadoi.cn/10.3969/j.issn.1672-2302.2005.02.008) | Adult | China | 17-81year | 2002.05-2002.12 | 36 | 0 | 36 |
| 49 | Clinical Analysis of 36 Severe Dengue Fever Cases in Guangzhou Area | Zhang FC et al | 2004 | [10.1111/j.1365-3156.2004.01295.x](https://doi.org/10.1111/j.1365-3156.2004.01295.x) | Adult | Thailand | 15-66year | 2001.01-2001.12 | 49 | 0 | 49 |
| 50 | Liver Impairment And Elevated Aminotransferase Levels Predict Severe Dengue In Vietnamese Children | RN Nguyen et al | 2019 | [10.1007/s12098-019-03040-0](https://doi.org/10.1007/s12098-019-03040-0) | Child | India | 5-10.3year | 2019.12-2020.12 | 22 | 0 | 22 |
| 51 | Severe Dengue and Associated Hemophagocytic Lymphohistiocytosis in PICU | Deepanjan B et al | 2023 | [10.7759/cureus.47606](https://doi.org/10.7759/cureus.47606) | Child | Vietnam | ＜15year | 2018.04-2020.05 | 69 | 0 | 69 |
| 52 | Risk Factors Associated With Death In Brazilian Children With Severe Dengue: A Case-Control Study | MRFC Branco et al | 2014 | [10.6061/clinics/2014(01)08](https://doi.org/10.6061/clinics/2014(01)08) | Child | Brazil | 0-12year | 2006.04-2007.12 | 77 | 0 | 77 |

Note: - indicates missing DOI information
