# Supplementary material for: Global estimation of dengue disability weights based on clinical manifestations data
Source: Infect Dis Poverty. 2025 Jun 9;14:44. doi: 10.1186/s40249-025-01317-5 (PMC12147332; doi:10.1186/s40249-025-01317-5)
Supplement: Supplementary file 4 — Supplementary Material 4: Quality assessment. [file 40249_2025_1317_MOESM4_ESM.docx]

**Supplementary file 4.** Quality assessment

| **No.** | **Type** | **Source** | **Author** | **Title** | **Define the source of information (survey, record review)** | **List inclusion and exclusion criteria for exposed and unexposed subjects (cases and controls) or refer to previous publications** | **Indicate period used for identifying patients** | **Indicate whether subjects were consecutive if not population-based** | **Indicate if evaluators of subjective components of study were masked to other aspects of the participants** | **Describe any assessments undertaken for quality assurance purposes (e.g.,test/retest of primary outcome measurements)** | **Explain any patient exclusions from analysis** | **Describe how confounding was assessed and/or controlled** | **If applicable, explain how missing data were handled in the analysis** | **Summarize patient response rates and completeness of data collection** | **Clarify what follow-up, if any, was expected and the percentage of patients for which incomplete data or follow-up was obtained** | **Quality evaluation** |
| --- | --- | --- | --- | --- | --- | --- | --- | --- | --- | --- | --- | --- | --- | --- | --- | --- |
| 1 | Adult | China | Qian ZX et al | Clinical Analysis of 110 Cases of Dengue Fever | Yes | Yes | Yes | Yes | Unclear | Yes | Yes | Unclear | Yes | Yes | Not Applicable | Eight |
| 2 | Adult | Saudi Arabia | Nasim A. Khan | Clinical profile and outcome of hospitalized patients during first outbreak of dengue in Makkah, Saudi Arabia | Yes | Yes | Yes | Yes | Unclear | Yes | Yes | Unclear | Yes | Yes | Not Applicable | Eight |
| 3 | Adult | Thailand | Aung KLL et al | Factors associated with severe clinical manifestation of dengue among adults in Thailand | Yes | Yes | Yes | Yes | Unclear | Yes | Yes | Unclear | Yes | Yes | Not Applicable | Eight |
| 4 | Adult | China | Kuo HJ et al | Analyses of clinical and laboratory characteristics of dengue adults at their hospital presentations based on the World Health Organization clinical-phase framework: Emphasizing risk of severe dengue in the elderly | Yes | Yes | Yes | Yes | Unclear | Yes | Yes | Unclear | Yes | Yes | Not Applicable | Eight |
| 5 | Adult | Vietnam | Walter R Taylor | Dengue in adults admitted to a referral hospital in Hanoi, Vietnam | Yes | Yes | Yes | Yes | Unclear | Yes | Yes | Unclear | Yes | Yes | Not Applicable | Eight |
| 6 | Adult | Pakistan | ASMA NAZEER | Dengue fever out break in Lahore, Pakistan. A clinical management experience | Yes | Yes | Yes | Yes | Unclear | Yes | Yes | Unclear | Yes | Yes | Not Applicable | Eight |
| 7 | Adult | China | Zheng XY | Clinical Characteristics and Nursing Care of 25 Elderly Patients with Dengue Fever | Yes | Yes | Yes | Yes | Unclear | Yes | Yes | Unclear | Yes | Yes | Not Applicable | Eight |
| 8 | Adult | Thailand | Thanachartwet V et al | Identification of clinical factors associated with severe dengue among Thai adults: a prospective study | Yes | Yes | Yes | Yes | Unclear | Yes | Yes | Unclear | Yes | Yes | Yes | Nine |
| 9 | Adult | India | Gursheen Kaur, | Look Out For Fever: Clinical Profile Of Dengue In Young Adults In A Tertiary Care Center In North India | Yes | Yes | Yes | Yes | Unclear | Yes | Yes | Unclear | Yes | Yes | Not Applicable | Eight |
| 10 | Adult | Thailand | Temprasertrudee S et al | A Multicenter Study of Clinical Presentations and Predictive Factors for Severe Manifestation of Dengue in Adults | Yes | Yes | Yes | Yes | Unclear | Yes | Yes | Unclear | Yes | Yes | Not Applicable | Eight |
| 11 | Adult | China | Wu HM et al | Analysis of Clinical Diagnosis and Treatment Characteristics of 68 Patients with Dengue Fever | Yes | Yes | Yes | Yes | Unclear | Yes | Yes | Unclear | Yes | Yes | Not Applicable | Eight |
| 12 | Adult | Tanzania | Noémie Boillat-Blanco, etal | Dengue fever in Dar es Salaam, Tanzania: Clinical features and outcome in populations of black and non-black racial category | Yes | Yes | Yes | Yes | Unclear | Yes | Yes | No | Yes | Yes | Yes | Nine |
| 13 | Adult | China | He AH et al | Analysis of Clinical Characteristics of 199 Dengue Fever Patients | Yes | Yes | Yes | Yes | Unclear | Yes | Yes | Unclear | Yes | Yes | Not Applicable | Eight |
| 14 | Adult | China | YangLing et al | The epidemic characteristics of a dengue fever outbreak in a certain university in Guangzhou in 2014 | Yes | Yes | Yes | Yes | Unclear | Yes | Yes | Unclear | Yes | Yes | Not Applicable | Eight |
| 15 | Adult | China | YuTao et al | Clinical Analysis of 89 Cases of Dengue Fever in 2014 | Yes | Yes | Yes | Yes | Unclear | Yes | Yes | Unclear | Yes | Yes | Not Applicable | Eight |
| 16 | Adult | China | Yong Ping Lin, | Clinical and epidemiological features of the 2014 large-scale dengue outbreak in Guangzhou city, China | Yes | Yes | Yes | Yes | Unclear | Yes | Yes | Unclear | Yes | Yes | Not Applicable | Eight |
| 17 | Adult | India | Padyana M et al | Clinical profile and outcome of dengue fever in multidisciplinary intensive care unit of a tertiary level hospital in india | Yes | Yes | Yes | Yes | Unclear | Yes | Yes | Unclear | Yes | Yes | Not Applicable | Eight |
| 18 | Adult | China | Ling YH et al | Analysis of Influencing Factors and Clinical Characteristics of Dengue Fever Course | Yes | Yes | Yes | Yes | Unclear | Yes | Yes | Unclear | Yes | Yes | Not Applicable | Eight |
| 19 | Adult | India | Sasmita Kumari Bisoyi, | Clinical profile of dengue fever at SCB Medical College and Hospital, Cuttack, Odisha | Yes | Yes | Yes | Yes | Unclear | Yes | Yes | Unclear | Yes | Yes | Yes | Nine |
| 20 | Adult | China | Bao XJ et al | Clinical Characteristics and Nursing Analysis of 34 Cases of Dengue Fever | Yes | Yes | Yes | Yes | Unclear | Yes | Yes | Unclear | Yes | Yes | Not Applicable | Eight |
| 21 | Adult | China | Ren Ze-Ze, | A survey of clinical and laboratory characteristics of the dengue fever epidemic from 2017 to 2019 in Zhejiang, China | Yes | Yes | Yes | Yes | Unclear | Yes | Yes | Unclear | Yes | Yes | Not Applicable | Eight |
| 22 | Adult | India | Pereira MS et al | Factors associated with severity of illness in patients with dengue fever in a tertiary care hospital in southern India | Yes | Yes | Yes | Yes | Unclear | Yes | Yes | Unclear | Yes | Yes | Not Applicable | Eight |
| 23 | Adult | Afghanistan | Mohammad Nadir Sahak | Dengue fever as an emerging disease in Afghanistan: Epidemiology of the first reported cases | Yes | Yes | Yes | Yes | Unclear | Yes | Yes | Unclear | Yes | Yes | Not Applicable | Eight |
| 24 | Adult | China | Chen QB et al | Analysis of Epidemiological and Clinical Characteristics of Dengue Fever in Anxi County in 2019 | Yes | Yes | Yes | Yes | Unclear | Yes | Yes | Unclear | Yes | Yes | Not Applicable | Eight |
| 25 | Adult | Bangladesh | Rafi A et al | Dengue epidemic in a non-endemic zone of Bangladesh: Clinical and laboratory profiles of patients | Yes | Yes | Yes | Yes | Unclear | Yes | Yes | Unclear | Yes | Yes | Yes | Nine |
| 26 | Adult | China | ZhouZhi et al | The Epidemiological Characteristics and Clinical Manifestations of Dengue Fever Epidemic in Chongqing City in 2019 | Yes | Yes | Yes | Yes | Unclear | Yes | Yes | Unclear | Yes | Yes | Not Applicable | Eight |
| 27 | Adult | China | Chen ST et al | Analysis of Clinical Characteristics of 201 Adult Dengue Fever Cases in Chongqing Liangjiang New Area | Yes | Yes | Yes | Yes | Unclear | Yes | Yes | Unclear | Yes | Yes | Not Applicable | Eight |
| 28 | Adult | China | Liu FW et al | Analysis of Clinical Data of 82 Patients with Dengue Fever | Yes | Yes | Yes | Yes | Unclear | Yes | Yes | Unclear | Yes | Yes | Not Applicable | Eight |
| 29 | Child | China | Liao LY et al | Clinical Analysis of 90 Cases of Dengue Fever in Children | Yes | Yes | Yes | Yes | Unclear | Yes | Yes | Unclear | Yes | Yes | Not Applicable | Eight |
| 30 | Child | China | Chen KQ | Clinical Analysis of 659 Cases of Dengue Fever in Children | Yes | Yes | Yes | Yes | Unclear | Yes | Yes | Unclear | Yes | Yes | Not Applicable | Eight |
| 31 | Child | China | Huang YZ et al | Clinical Analysis of 331 Cases of Dengue Fever in Children | Yes | Yes | Yes | Yes | Unclear | Yes | Yes | Unclear | Yes | Yes | Not Applicable | Eight |
| 32 | Child | China | Mei FS et al | Clinical Analysis of 70 Cases of Dengue Fever in Children | Yes | Yes | Yes | Yes | Unclear | Yes | Yes | Unclear | Yes | Yes | Not Applicable | Eight |
| 33 | Child | China | ChenChi et al | Clinical Analysis of 210 Cases of Dengue Fever in Children | Yes | Yes | Yes | Yes | Unclear | Yes | Yes | Unclear | Yes | Yes | Not Applicable | Eight |
| 34 | Child | China | Feng LX | Clinical Analysis of 454 Cases of Dengue Fever in Children | Yes | Yes | Yes | Yes | Unclear | Yes | Yes | Unclear | Yes | Yes | Not Applicable | Eight |
| 35 | Child | China | Yu TT et al | Clinical Characteristics of Dengue Fever Cases in Children during the 2014 Dengue Outbreak in Guangzhou City | Yes | Yes | Yes | Yes | Unclear | Yes | Yes | Unclear | Yes | Yes | Not Applicable | Eight |
| 36 | Child | China | Wang YL et al | Clinical Analysis of 78 Cases of Dengue Fever in Children | Yes | Yes | Yes | Yes | Unclear | Yes | Yes | Unclear | Yes | Yes | Not Applicable | Eight |
| 37 | Child | China | LiuWei et al | Analysis of Clinical Characteristics and Laboratory Tests of Dengue Fever Patients among Children in Guangzhou City | Yes | Yes | Yes | Yes | Unclear | Yes | Yes | Unclear | Yes | Yes | Not Applicable | Eight |
| 38 | Child | China | HuDan et al | Analysis of Clinical Characteristics of 34 Cases of Dengue Fever in Infants and Young Children | Yes | Yes | Yes | Yes | Unclear | Yes | Yes | Unclear | Yes | Yes | Not Applicable | Eight |
| 39 | Child | China | Cai XP et al | Clinical Analysis of 33 Cases of Dengue Fever in Children | Yes | Yes | Yes | Yes | Unclear | Yes | Yes | Unclear | Yes | Yes | Not Applicable | Eight |
| 40 | Child | Sri Lanka | U Jayarajah, | Clinical and Biochemical Characteristics of Dengue Infections in Children From Sri Lanka | Yes | Yes | Yes | Yes | Unclear | Yes | Yes | Unclear | Yes | Yes | Not Applicable | Eight |
| 41 | Child | Jamaica | AM Lue | Severity and Outcomes of Dengue in Hospitalized Jamaican Children in 2018-2019 During an Epidemic Surge in the Americas | Yes | Yes | Yes | Yes | Unclear | Yes | Yes | Unclear | Yes | Yes | Yes | Nine |
| 42 | Child | China | GanYun et al | Clinical Analysis of Dengue Fever in Children | Yes | Yes | Yes | Yes | Unclear | Yes | Yes | Unclear | Yes | Yes | Not Applicable | Eight |
| 43 | Adult | Thailand | Ole Wichmann | Risk factors and clinical features associated with severe dengue infection in adults and children during the 2001 epidemic in Chonburi, Thailand | Yes | Yes | Yes | Yes | Unclear | Yes | Yes | Unclear | Yes | Yes | Not Applicable | Eight |
| 44 | Adult | Thailand | Vipa Thanachartwet et al | Identification of clinical factors associated with severe dengue among Thai adults: A prospective study | Yes | Yes | Yes | Yes | Unclear | Yes | Yes | Unclear | Yes | Yes | Yes | Nine |
| 45 | Adult | Saudi Arabia | Moustafa A. Hegazi et al | Risk Factors and Predictors of Severe Dengue in Saudi Population in Jeddah, Western Saudi Arabia: A Retrospective Study. | Yes | Yes | Yes | Yes | Unclear | Yes | Yes | Unclear | Yes | Yes | Not Applicable | Eight |
| 46 | Adult | China | Cai WP et al | Clinical Characteristics and Therapeutic Experience of 121 Adult Patients with Severe Dengue Fever | Yes | Yes | Yes | Yes | Unclear | Yes | Yes | Unclear | Yes | Yes | Not Applicable | Eight |
| 47 | Adult | China | Lu YH et al | Clinical Analysis of 65 Severe Dengue Fever Cases in Guangzhou City | Yes | Yes | Yes | Yes | Unclear | Yes | Yes | Unclear | Yes | Yes | Not Applicable | Eight |
| 48 | Adult | China | Ye QX et al | Nursing care of 16 patients with severe dengue fever | Yes | Yes | Yes | Yes | Unclear | Yes | Yes | Unclear | Yes | Yes | Not Applicable | Eight |
| 49 | Adult | China | Zhang FC et al | Clinical Analysis of 36 Severe Dengue Fever Cases in Guangzhou Area | Yes | Yes | Yes | Yes | Unclear | Yes | Yes | Unclear | Yes | Yes | Not Applicable | Eight |
| 50 | Child | Vietnam | RN Nguyen et al | Liver Impairment And Elevated Aminotransferase Levels Predict Severe Dengue In Vietnamese Children | Yes | Yes | Yes | Yes | Unclear | Yes | Yes | Unclear | Yes | Yes | Yes | Nine |
| 51 | Child | India | Deepanjan Bhattacharya et al | Severe Dengue and Associated Hemophagocytic Lymphohistiocytosis in PICU | Yes | Yes | Yes | Yes | Unclear | Yes | Yes | Unclear | Yes | Yes | Not Applicable | Eight |
| 52 | Child | Brazil | MRFC Branco et al | Risk Factors Associated With Death In Brazilian Children With Severe Dengue: A Case-Control Study | Yes | Yes | Yes | Yes | Unclear | Yes | Yes | Unclear | Yes | Yes | Not Applicable | Eight |

Notes: The assessment consists of 11 items, each answered with "yes," "no," "unclear," or "not applicable". "Yes" responses were scored as 1 point, while "no," "unclear," or "not applicable" responses received 0 points. Studies scoring 0-3 points were considered low quality, 4-7 points medium quality, and 8-11 points high quality.
