# Supplementary material for: Global estimation of dengue disability weights based on clinical manifestations data
Source: Infect Dis Poverty. 2025 Jun 9;14:44. doi: 10.1186/s40249-025-01317-5 (PMC12147332; doi:10.1186/s40249-025-01317-5)
Supplement: Supplementary file 5 — Supplementary Material 5: Disability Weights and Log-Normal Distribution of Dengue Clinical Manifestations, odds ratio (OR) and Chi-Square Test Results for Population Difference. [file 40249_2025_1317_MOESM5_ESM.docx]

**Supplementary file 5.** Disability Weights and Log-Normal Distribution of Dengue Clinical Manifestations, odds ratio (*OR*) and Chi-Square Test Results for Population Differences.

| **Signs and symptoms** | **Source item** | **Reference source** | **DW mean** | **2.5%** | **97.5%** | **Log_DW mean** | **Log_DW mean SD** | ***OR* (95% *CI*)** | **Chi-Square value**  **(*P* value)** |
| --- | --- | --- | --- | --- | --- | --- | --- | --- | --- |
| Fever | High fever | GBD 2021 | 0.133 | 0.088 | 0.190 | -2.016 | 0.1963488 | 0.00 (0.00, 0.05) | 73.44 (<0.0001) |
| Weak | Feeling weak, causing some difficulty in daily activities | GBD 2021 | 0.051 | 0.032 | 0.074 | -2.978 | 0.2368227 | 4.95 (4.12, 5.98) | 349.25 (<0.0001) |
| Decreased appetite | Loss of appetite | GBD 2021 | 0.011 | 0.005 | 0.021 | -4.510 | 0.3087652 | 0.12 (0.11, 0.14) | 1111.54 (<0.0001) |
| Nausea | Nausea, not affecting daily activities | GBD 2021 | 0.011 | 0.005 | 0.021 | -4.510 | 0.3087652 | 1.43 (1.23, 1.67) | 20.82 (<0.0001) |
| Vomiting | Nausea with vomiting | GBD 2021 | 0.011 | 0.005 | 0.021 | -4.510 | 0.3087652 | 2.63 (2.23, 3.11) | 144.48 (<0.0001) |
| Abdominal pain | Some abdominal pain, causing nausea | Finkelstein  et al. 2007 | 0.060 | - | - | -2.813 | - | 0.99 (0.88, 1.12) | 0.01 (0.9248) |
| Diarrhea | Mild diarrhea | GBD 2021 | 0.074 | 0.049 | 0.104 | -2.603 | 0.1801775 | 2.95 (2.37, 3.72) | 100.67 (<0.0001) |
| Headache | Mild headache | GBD 2021 | 0.067 | 0.041 | 0.103 | -2.705 | 0.2464043 | 1.76 (1.61, 1.93) | 154.81 (<0.0001) |
| Muscular pain | Mild musculoskeletal issues | GBD 2021 | 0.023 | 0.013 | 0.037 | -3.772 | 0.2761415 | 9.18 (8.17, 10.33) | 1723.73 (<0.0001) |
| Joint pain | Bone pain fever (using the mild rheumatoid arthritis DW0.117) | GBD 2021 | 0.117 | 0.080 | 0.163 | -2.145 | 0.1876743 | 1.05 (0.91, 1.22) | 0.45 (0.5003) |
| Skin flush | - | GBD 2021 | 0.011 | 0.005 | 0.021 | -4.510 | 0.3087652 | 0.03 (0.01, 0.05) | 381.70 (<0.0001) |
| Conjunctival injection | - | GBD 2017 | 0.052 | 0.034 | 0.076 | -2.955 | 0.2419331 | 0.07 (0.04, 0.10) | 347.92 (<0.0001) |
| Skin rash | Bruising, petechiae | GBD 2021 | 0.058 | 0.035 | 0.090 | -2.845 | 0.2699675 | 1.62 (1.46, 1.78) | 92.55 (<0.0001) |
| Subcutaneous hemorrhage | - | GBD 2017 | 0.052 | 0.034 | 0.076 | -2.955 | 0.2419331 | 0.58 (0.42, 0.81) | 11.27 (0.0008) |
| Gingival bleeding | Slight gum bleeding | GBD 2017 | 0.007 | 0.003 | 0.014 | -4.969 | 0.3524474 | 4.69 (2.52, 9.67) | 27.50 (<0.0001) |
| Epistaxis | - | GBD 2017 | 0.052 | 0.034 | 0.076 | -2.955 | 0.2419331 | 0.21 (0.15, 0.28) | 124.39 (<0.0001) |
| Ecchymosis | Bruising, petechiae | GBD 2021 | 0.058 | 0.035 | 0.090 | -2.845 | 0.2699675 | 5.03 (2.44, 12.00) | 21.61 (<0.0001) |
| Gross hematuria | Kidney damage | GBD 2021 | 0.571 | 0.398 | 0.725 | -0.561 | 0.108623 | 0.13 (0.05, 0.31) | 29.22 (<0.0001) |
| Gastrointestinal bleeding | Gastric bleeding, vomiting blood and feeling nauseous | GBD 2021 | 0.325 | 0.209 | 0.462 | -1.124 | 0.149182 | 16.22 (5.40, 79.88) | 40.74 (<0.0001) |
| Thoracoabdominal hemorrhage | - | GBD 2021 | 0.325 | 0.209 | 0.462 | -1.124 | 0.149182 | - | 1.58 (0.2085) |
| Vaginal bleeding | - | GBD 2021 | 0.325 | 0.209 | 0.462 | -1.124 | 0.149182 | - | 4.32 (0.0376) |
| Intracranial hemorrhage | Acute, not affecting mobility | GBD 2021 | 0.019 | 0.010 | 0.032 | -3.965 | 0.216695 | - | - |
| Lymphadenectasis | Lymphadenopathy | GBD 2017 | 0.109 | 0.073 | 0.154 | -2.220 | 0.157248 | 0.04 (0.03, 0.06) | 1033.72 (<0.0001) |
| Hepatomegaly | Mild abdominal pelvic issues | GBD 2017 | 0.011 | 0.005 | 0.021 | -4.510 | 0.3087652 | 0.41 (0.31, 0.54) | 43.96 (<0.0001) |
| Splenomegaly | Splenomegaly | Olveda et al.1983 | 0.070 | - | - | -2.659 | - | 0.23 (0.13, 0.41) | 31.59 (<0.0001) |
| Shock | - | GBD 2021 | 0.252 | - | - | -1.379 | - | 1.13 (0.70, 1.87) | 0.12 (0.7342) |
| Cardiac function damage | Acute myocarditis/acute heart failure, etc. | GBD 2004 | 0.252 | - | - | -1.379 | - | - | - |
| Brain-related injury | Complicated with encephalitis/meningitis, etc. | GBD 2021 | 0.133 | 0.088 | 0.190 | -2.016 | 0.1963488 | - | 7.13 (0.0076) |
| Lung outcomes | Respiratory tract infection episodes, etc. | GBD 2004 | 0.279 | - | - | -1.276 | - | - | 9.96 (0.0016) |
| Liver function damage | Acute liver injury/liver failure, etc. | Finkelstein et al. 2007 | 0.330 | - | - | -1.108 | - | 0.15 (0.11, 0.21) | 197.82 (<0.0001) |
| Renal function damage | Combined kidney damage/acute renal failure, etc. | GBD 2021 | 0.571 | 0.398 | 0.725 | -0.561 | 0.1485821 | - | 7.13 (0.0076) |

Notes: -: Not Available / Not Applicable.
