# Supplementary material for: Global estimation of dengue disability weights based on clinical manifestations data
Source: Infect Dis Poverty. 2025 Jun 9;14:44. doi: 10.1186/s40249-025-01317-5 (PMC12147332; doi:10.1186/s40249-025-01317-5)
Supplement: Supplementary file 6 — Supplementary Material 6: The global distribution of dengue-related YLDs across countries in 2021. [file 40249_2025_1317_MOESM6_ESM.docx]

**Supplementary file 6.** The global distribution of dengue-related YLDs across countries in 2021.

| **Continent** | **Country** | **2021 Dengue Fever YLD** |
| --- | --- | --- |
| Africa | Angola | 863.49 |
|  | Benin | 860.89 |
|  | Burkina Faso | 411.81 |
|  | Burundi | 28.79 |
|  | Cameroon | 1339.44 |
|  | Cape Verde | 541.23 |
|  | Central African Republic | 169.41 |
|  | Chad | 100.85 |
|  | Comoros | 1103.41 |
|  | Congo | 205.88 |
|  | Cote d’Ivoire | 728.69 |
|  | Democratic Republic of Congo | 1698.79 |
|  | Djibouti | 95.94 |
|  | Egypt | 337.43 |
|  | Equatorial Guinea | 22.61 |
|  | Eritrea | 8.62 |
|  | Ethiopia | 205.72 |
|  | Gabon | 102.65 |
|  | Gambia | 139.51 |
|  | Ghana | 3073.37 |
|  | Guinea | 171.33 |
|  | Guinea-Bissau | 105.98 |
|  | Kenya | 2123.21 |
|  | Liberia | 376.37 |
|  | Madagascar | 122.02 |
|  | Malawi | 76.42 |
|  | Mali | 178.56 |
|  | Mauritania | 23.42 |
|  | Mauritius | 155.12 |
|  | Mozambique | 449.23 |
|  | Niger | 114.63 |
|  | Nigeria | 21485.00 |
|  | Rwanda | 19.47 |
|  | Sao Tome and Principe | 21.67 |
|  | Senegal | 137.80 |
|  | Seychelles | 158.87 |
|  | Sierra Leone | 592.03 |
|  | Somalia | 109.47 |
|  | South Sudan | 1.16 |
|  | Sudan | 89.50 |
|  | Tanzania | 121.72 |
|  | Togo | 546.44 |
|  | Uganda | 271.65 |
|  | Zambia | 102.96 |
|  | Zimbabwe | 13.58 |
| Asia | Afghanistan | 7.85 |
|  | Bangladesh | 8748.81 |
|  | Bhutan | 4.90 |
|  | Brunei | 7.17 |
|  | Cambodia | 527.26 |
|  | China | 334.25 |
|  | East Timor | 39.05 |
|  | India | 352468.54 |
|  | Indonesia | 31097.01 |
|  | Jordan | 22.73 |
|  | Kuwait | 32.12 |
|  | Laos | 296.67 |
|  | Lebanon | 22.21 |
|  | Malaysia | 10199.53 |
|  | Maldives | 323.60 |
|  | Myanmar | 555.25 |
|  | Nepal | 3460.01 |
|  | Oman | 12.57 |
|  | Pakistan | 32856.81 |
|  | Palestine | 12.78 |
|  | Philippines | 15779.54 |
|  | Saudi Arabia | 22.01 |
|  | Singapore | 6062.51 |
|  | Sri Lanka | 5827.16 |
|  | Syria | 6.49 |
|  | Thailand | 5207.14 |
|  | Vietnam | 13792.71 |
|  | Yemen | 100.90 |
| North America | Antigua and Barbuda | 0.56 |
|  | Bahamas | 66.32 |
|  | Barbados | 72.03 |
|  | Belize | 25.82 |
|  | Costa Rica | 1591.26 |
|  | Cuba | 238.37 |
|  | Dominica | 1.72 |
|  | Dominican Republic | 739.46 |
|  | El Salvador | 1213.78 |
|  | Grenada | 4.30 |
|  | Guatemala | 455.63 |
|  | Haiti | 667.76 |
|  | Honduras | 1113.01 |
|  | Jamaica | 54.38 |
|  | Mexico | 17533.63 |
|  | Nicaragua | 941.13 |
|  | Panama | 628.12 |
|  | Puerto Rico | 713.38 |
|  | Saint Kitts and Nevis | 0.49 |
|  | Saint Lucia | 5.44 |
|  | Saint Vincent and the Grenadines | 4.81 |
|  | Trinidad and Tobago | 100.82 |
|  | United States | 17.20 |
|  | United States Virgin Islands | 2.01 |
| Oceania | American Samoa | 14.22 |
|  | Australia | 230.53 |
|  | Cook Islands | 0.24 |
|  | Fiji | 192.41 |
|  | Kiribati | 92.47 |
|  | Marshall Islands | 72.42 |
|  | Federated States of Micronesia | 5.55 |
|  | Nauru | 1.47 |
|  | Niue | 0.02 |
|  | Northern Mariana Islands | 4.50 |
|  | Palau | 7.15 |
|  | Papua New Guinea | 42.63 |
|  | Samoa | 14.80 |
|  | Solomon Islands | 92.51 |
|  | Tonga | 191.50 |
|  | Tuvalu | 0.70 |
|  | Vanuatu | 30.94 |
| South America | Argentina | 1001.28 |
|  | Bolivia | 1035.38 |
|  | Brazil | 160741.80 |
|  | Colombia | 9212.45 |
|  | Ecuador | 1177.11 |
|  | Guyana | 22.47 |
|  | Paraguay | 2251.70 |
|  | Peru | 2682.46 |
|  | Suriname | 21.42 |
|  | Venezuela | 3383.74 |

Notes: YLD represents Years Lived with Disability. The countries within each continent and region are listed in alphabetical order.
