# Supplementary material for: Global estimation of dengue disability weights based on clinical manifestations data
Source: Infect Dis Poverty. 2025 Jun 9;14:44. doi: 10.1186/s40249-025-01317-5 (PMC12147332; doi:10.1186/s40249-025-01317-5)
Supplement: Supplementary file 7 — Supplementary Material 7: The global distribution of dengue-related clinical English-language literature. [file 40249_2025_1317_MOESM7_ESM.docx]

**Supplementary file 7.** The global distribution of dengue-related clinical English-language literature.

| **Continent** | **Country** | **The number of literatures** |
| --- | --- | --- |
| Africa | Angola | 1 |
|  | Burkina Faso | 2 |
|  | Cote d'Ivoire | 1 |
|  | Egypt | 1 |
|  | Ethiopia | 1 |
|  | Nigeria | 1 |
|  | Tanzania | 1 |
|  | Uganda | 1 |
| Asia | Afghanistan | 1 |
|  | Bangladesh | 13 |
|  | Bhutan | 1 |
|  | Brunei | 1 |
|  | Cambodia | 3 |
|  | China | 19 |
|  | Filipino | 1 |
|  | India | 80 |
|  | Indonesia | 18 |
|  | Iran | 2 |
|  | Japan | 23 |
|  | Korea | 2 |
|  | Kuwait | 1 |
|  | Laos | 1 |
|  | Malaysia | 24 |
|  | Nepal | 7 |
|  | Oman | 1 |
|  | Pakistan | 39 |
|  | Philippines | 5 |
|  | Saudi Arabia | 12 |
|  | Singapore | 19 |
|  | Sri Lanka | 17 |
|  | Thailand | 43 |
|  | Vietnam | 16 |
|  | Yemen | 2 |
| Europe | Austria | 3 |
|  | Belgium | 4 |
|  | Czech Republic | 1 |
|  | France | 29 |
|  | Germany | 4 |
|  | Italy | 8 |
|  | Netherlands | 5 |
|  | Portugal | 3 |
|  | Spain | 4 |
|  | Turkey | 3 |
|  | United Kingdom | 14 |
| North America | Barbados | 8 |
|  | Cuba | 10 |
|  | El Salvador | 2 |
|  | Haiti | 1 |
|  | Honduras | 2 |
|  | Jamaica | 4 |
|  | Mexico | 14 |
|  | Nicaragua | 2 |
|  | Trinidad and Tobago | 1 |
|  | United States | 34 |
| Oceania | Australia | 17 |
|  | Fiji | 1 |
|  | Papua New Guinea | 1 |
| South America | Argentina | 1 |
|  | Brazil | 24 |
|  | Colombia | 11 |
|  | Ecuador | 2 |
|  | Guyana | 1 |
|  | Paraguay | 1 |
|  | Peru | 3 |
|  | Uruguay | 1 |
|  | Venezuela | 1 |

Notes: The countries within each continent and region are listed in alphabetical order.
